# TNFα secreted by glioma associated macrophages promotes endothelial activation and resistance against anti-angiogenic therapy

**DOI:** 10.1186/s40478-021-01163-0

**Published:** 2021-04-14

**Authors:** Qingxia Wei, Olivia Singh, Can Ekinci, Jaspreet Gill, Mira Li, Yasin Mamatjan, Shirin Karimi, Severa Bunda, Sheila Mansouri, Kenneth Aldape, Gelareh Zadeh

**Affiliations:** 1 MacFeeters Hamilton Centre for Neuro-Oncology Research, Princess Margaret Cancer Centre, University Health Network, Toronto, ON M5G 1L7 Canada; 2 University Health Network, Toronto Western Hospital, Toronto, Canada; 3 Division of Neurosurgery, Department of Surgery, The University of Toronto, 399 Bathurst Street, West Wing 4-439, Toronto, ON M5T 2S8 Canada

**Keywords:** Glioblastoma, Glioma-associated macrophages, Endothelial cells activation, TNFα, Anti-angiogenic therapy

## Abstract

One of the most prominent features of glioblastoma (GBM) is hyper-vascularization. Bone marrow-derived macrophages are actively recruited to the tumor and referred to as glioma-associated macrophages (GAMs) which are thought to provide a critical role in tumor neo-vascularization. However, the mechanisms by which GAMs regulate endothelial cells (ECs) in the process of tumor vascularization and response to anti-angiogenic therapy (AATx) is not well-understood. Here we show that GBM cells secrete IL-8 and CCL2 which stimulate GAMs to produce TNFα. Subsequently, TNFα induces a distinct gene expression signature of activated ECs including VCAM-1, ICAM-1, CXCL5, and CXCL10. Inhibition of TNFα blocks GAM-induced EC activation both in vitro and in vivo and improve survival in mouse glioma models. Importantly we show that high TNFα expression predicts worse response to Bevacizumab in GBM patients. We further demonstrated in mouse model that treatment with B20.4.1.1, the mouse analog of Bevacizumab, increased macrophage recruitment to the tumor area and correlated with upregulated TNFα expression in GAMs and increased EC activation, which may be responsible for the failure of AATx in GBMs. These results suggest TNFα is a novel therapeutic that may reverse resistance to AATx. Future clinical studies should be aimed at inhibiting TNFα as a concurrent therapy in GBMs.

**Supplementary Information:**

The online version contains supplementary material available at 10.1186/s40478-021-01163-0.

## Introduction

Glioblastoma (GBM) is the most common and lethal primary adult brain tumor. Despite maximal surgical resection, chemotherapy, and radiation, the prognosis for GBM patients remains very poor with a median survival time of only 12–15 months [34]. These highly invasive and vascularized tumors inevitably escape treatment and recur in nearly all cases, emphasizing the need for a better understanding of the biology of these aggressive tumors.

Given that GBMs are a highly vascular tumor, anti-angiogenic therapies (AATx) have held considerable promise. However, inhibitors targeting the key angiogenic regulator, vascular endothelial growth factor (VEGF), have shown a lack of efficacy in the clinic. Two phase III clinical trials targeting VEGF showed that Bevacizumab failed to improve overall survival (OS) in GBM patients, diminishing the enthusiasm for AATx in these tumors [10, 15]. Therefore, a greater understanding of the underlying molecular mechanisms that promote angiogenesis is critical for the development of more effective AATx in GBMs.

A key histological characteristic of GBMs is the presence of pathologically altered vessels that are torturous, hyper-dilated, and leaky [3, 19]. Within these vessels, tumor endothelial cells (ECs) exhibit an activated or pro-inflammatory state characterized by increased expression of surface adhesion molecules such as VCAM1 and ICAM1, which facilitate the recruitment and attachment of leukocytes to the vessel wall [13]. It is posited that these bound leukocytes then transmigrate across the endothelial layer into the brain to contribute to the neuro-inflammatory response. Accumulating evidence suggests this chronic inflammatory state can lead to endothelial dysfunction and aberrant angiogenesis that may drive evasion and resistance to AATx [11, 30]. The underlying mechanisms by which ECs are activated, specifically factors and biological processes that result in EC activation in GBM remains unknown.

Tumor angiogenesis and growth are highly dependent upon host cell populations within the tumor microenvironment [28, 31]. Our laboratory, among other groups, have shown that bone marrow-derived macrophages are actively recruited to perivascular regions of tumors, where they remain closely associated with vessels and are thought to play important roles in supporting tumor vasculature [21, 29]. A deeper understanding into the mechanism by which surrounding tumor cells lead to “re-programming” of Glioma-associated macrophages(GAMs) and how this consequently influences the tumor vasculature could provide necessary insights for the design of effective therapeutics.

Here we explore the mechanisms by which GAMs induce activation of ECs, which result in aberrant GBM angiogenesis and contributes to resistance to AATx. We demonstrate that glioma cell-derived IL8 and CCL2 stimulate GAMs to secrete TNFα and promote EC activation. In turn, TNFα inhibition prevents EC activation and leads significant improvement in survival of GBM mouse xenograft model. More importantly, we validate these findings in a clinical cohort showing that TNFα is a predictor of OS and response to AATx in GBM patients. Collectively, these results provide compelling evidence to further explore inhibition of TNFα concurrent with AATx as a novel therapeutic strategy.

## Materials and methods

### Cells

RAW264.7, U87, U118, U251, A172 and U973 were obtained from the American Type Culture Collection and maintained in Dulbecco’s modified Eagle’s medium (DMEM) (Invitrogen) or Roswell Park Memorial Institute(RPMI)-1640 medium(Sigma-Aldrich) for U973 cells supplemented with 10% heat-inactivated fetal bovine serum (FBS) (Wisent). Non-immortalized normal human astrocytes (NHA) were obtained from Lonza and maintained in astrocyte growth medium (AGM) (Lonza). Human neural stem cells-H9 derived (hNSC-h9) were obtained from Gibco. Glioma stem cells (GSC28 and GSC267) were derived from freshly operated tumor samples from GBM patients at the University of Texas MD Anderson Cancer Center as per guidelines set by institutional review board guidelines. Each patient provided written informed consent for tumor tissues and this study was conducted under protocol LAB03-0687, which was approved by the institutional review board of the University of Texas M. D. Anderson Cancer Center [38]. GSC 28 and GSC 267 were characterized as they are both IDH WT GBM that belong to the Mesenchymal (expressing YKL40, SERPINE1, TIMP1, and TGFBI) subtype. Patient information such as age, sex was not disclosed in the original paper [4]. Human umbilical vein endothelial cells (HUVEC) were obtained from Lonza and maintained in endothelial growth media 2 (EGM-2) and used between passage 2–8. Human cerebral endothelial cell line, hCMEC/D3, was obtained from Millipore and grown on collagen-coated plates in EGM-2 medium. GL261 cells were obtained from the National Cancer Institute (NCI) at Frederick and maintained in RPMI medium supplemented with 10% heat-inactivated FBS. Normal mouse astrocytes (NMA) were isolated from C57BL/6 mice as previously described [33] and grown in DMEM supplemented with 10% heat-inactivated FBS (Wisent), 16 mM HEPES, 1 × Non-Essential Amino Acids (NEAA) (ThermoFisher), 2 mM glutamine (Wisent), 2.5 µg/mL penicillin/streptomycin (Wisent), and 50 µg/mL gentamicin (Sigma). All cell lines were grown at 37 °C in a humidified 5% CO_2_ atmosphere and tested negative for mycoplasma.

### Preparation of glioma-associated macrophage conditioned medium (GAM-CM)

NHA, U87, U118, U251, A172, NSC, GSC28 or GSC267 cells were seeded in 10 cm plates. When cells were 50% confluent, the medium was replaced with fresh DMEM supplemented with 10% HI-FBS. Twenty-four hours later the conditioned medium was harvested from the cells, centrifuged at 200xg for 5 min to remove any cells, and supernatant was transferred onto RAW264.7 cells. RAW264.7 cells were incubated with conditioned medium for 24 h, washed twice with PBS, and replaced with EBM-2 supplemented with 0.5% HI-FBS. Twenty-four hours later the medium was collected, centrifuged as above, and the resulting supernatant (referred to as GAM-CM), was used for subsequent experiments.

### Angiogenesis RT-PCR array

HUVEC cells were treated with EBM-2 supplemented with 0.5% HI-FBS or GAM-CM (prepared as described above) for 24 h at 37 °C. Cells were washed once with 1x PBS and total RNA was extracted using Trizol (Invitrogen) according to the manufacturer’s instructions. For the synthesis of cDNA, 1 μg of RNA was reverse transcribed using QuantiTech reverse transcription kit (Qiagen). Quantitative PCR was performed in an 8 µL reaction volume in a 384-well plate containing the following: 7.5 ng cDNA, 1 × FastStart Universal SYBR green master mix (Rox) (Sigma), 0.31 µM forward and reverse primers. Reactions conditions were as follows: 95 °C (2 min hold); 95 °C (3 s), 60 °C (25 s) for 40 cycles; followed by a standard melt curve cycle. Primer sets are listed in Additional file 1: Supplemental Table 1. All reactions were performed in triplicate. Values were normalized to *ACTB* and *RPL30* and expressed as mean ± max/min (95% confidence interval).

**Table 1:** Dual IHC with CD68 and TNFα or TNFα and VCAM1 on pretreated human IDH-wt GBM sections

Pathology	Avastin (A) or Untreated (U)	CD68 positive cells	TNFa positive cells	% of double positive cells	Tissue area (μm^2^)	CD68 positive/mm2	% TNFa Positive Cells/CD68 positive/mm^2^	VCAM1	Survival from start of Avastin (days)
S10-10131	U	11,724	10,305	87.896622	50,922,572	230.2318901	0.381774314	High	
S10-1813	U	10,403	297	2.854946	86,029,352	120.9238447	0.023609454	Low	
S10-18509	U	13,843	11,179	80.755615	221,351,008	62.53868065	1.291290673	High	
S10-18918	U	7416	1082	14.590075	184,785,120	40.13310163	0.363542174	Med	
S10-20729	U	1723	818	47.475334	223,205,936	7.719328755	6.150189415	High	
S10-23850	U	4105	4034	98.270401	65,117,496	63.0398933	1.558860522	High	
S10-24725	U	2858	2793	97.725685	16,088,890	177.6381093	0.550139187	High	
S10-25121	U	1657	19	1.146651	121,491,016	13.63886857	0.084072296	low	
S10-25659	U	11,966	6770	56.576969	18,454,342	648.4110894	0.087254783	Low	
S10-2928	U	7170	4231	59.009762	62,469,184	114.7765913	0.514127152	Med	
S10-31365	U	8253	4636	56.173512	71,736,504	115.0460301	0.488269886	low	
S10-32073	U	17,689	6975	39.431286	125,374,008	141.0898501	0.279476418	low	
S10-32286	U	19,867	6373	32.07832	71,390,272	278.2872154	0.115270549	Med	
S10-33315	U	1131	398	35.190098	228,310,704	4.953775623	7.10369235	High	
S10-33655	U	3338	852	25.524265	181,316,576	18.40978952	1.386450669	High	
S10-36172	U	2921	2837	97.124275	13,749,701	212.4409833	0.457182383	High	
S10-37332	U	5289	3948	74.645493	72,317,608	73.13571544	1.020643506	High	
S10-37372	U	8600	5705	66.337212	88,101,760	97.61439499	0.679584318	Med	
S10-37487	U	5529	3230	58.419243	82,281,136	67.19644707	0.869379938	Med	
S10-38393	U	26,935	13,018	48.331169	81,640,520	329.9219554	0.146492733	Med	
S10-41235	U	18,022	10,180	56.486515	105,453,928	170.8992765	0.330525185	Med	
S10-5238	U	19,134	4786	25.013065	147,458,384	129.7586443	0.192766078	Low	
S10-6120	U	14,592	14,338	98.259323	14,052,854	1038.365588	0.094628832	Low	
S10-6519	U	1670	1644	98.443115	3,186,010.75	524.1664674	0.187808876	Low	
S10-8682	U	346	45	13.00578	242,124,560	1.429016536	9.101210289	High	
S10-27805	A	8556	4975	58.146332	209,370,352	40.8653848	1.422874941	Med	334
S09-12484	A	7436	0	0	101,027,248	73.60390535	0	Low	708
S11-33469	A	32,588	4493	13.787284	138,144,512	235.897898	0.058445981	Low	497
S11-37524	A	12,304	6065	49.292912	70,076,544	175.579435	0.280744223	High	85
S11-37558	A	36,049	1530	4.244223	300,741,824	119.8669328	0.035407788	Low	289
S12-13992	A	10,632	825	7.759593	206,245,344	51.55025463	0.150524824	Low	295
S12-44046	A	1867	509	27.262989	129,823,864	14.3810232	1.895761423	High	194
S12-49013	A	3578	840	23.476803	253,127,344	14.13517775	1.660877806	High	116
S12-55823	A	20,895	15,731	75.285957	65,120,496	320.8667207	0.234633111	Med	
S12-65416	A	14,518	6556	45.157734	59,637,560	243.4371896	0.185500556	Low	409
S12-78023	A	986	0	0	292,213,728	3.374242568	0	Low	97
S13-23668	A	8512	2619	30.768328	88,663,336	96.00360627	0.320491377	Med	152
S13-27592	A	708	482	68.079094	36,016,252	19.65779227	3.46321159	High	80
S14-35506	A	5734	4572	79.734917	57,075,904	100.4627101	0.793676747	High	69
Average		10,013.94872	4351.025641	47.53233069	117,581,891	158.7550979	1.127189547		

### Immunoblotting

Cells were washed with PBS and lysed in cold EBC buffer (50 mM Tris [pH 8.0], 120 mM NaCl, 0.5% NP-40) supplemented with a cocktail of protease and phosphatase inhibitors (Roche). Cell lysates were sonicated for 5 s followed by centrifugation at 14,000 rpm at 4 °C for 10 min to remove cell debris. For analysis of conditioned medium, media was concentrated by centrifugation through an Amicon Ultra centrifugal filter with a membrane nominal molecular weight limit (NMWL) of 3 kDa. Protein concentrations were determined by BCA method using Pierce BCA Protein Assay (ThermoScientific). Whole cell extracts were combined with sodium dodecyl sulfate (SDS)-containing sample buffer (2% SDS, 10% glycerol, 0.01% bromophenol blue, 62.5 mM Tris–HCl (pH 6.8), 0.1 M DTT), incubated at 95 °C for 5 min, and separated by sodium dodecyl sulfate–polyacrylamide gel electrophoresis (SDS-PAGE). Proteins were transferred to polyvinylidene difluoride (PVDF) for immunoblotting (Bio-Rad Laboratories). The membrane was subsequently blocked in TBST (10 mM Tris pH 8.0, 150 mM NaCl, 0.05% Tween-20) containing 4% skim milk powder for 1 h at room temperature, washed with TBST, and then incubated overnight at 4 °C with primary antibody diluted in TBST. The following antibodies were used: anti-VCAM1 (AbCam, ab134047), anti-ICAM1 (AbCam, ab53013), anti-α-Tubulin (Cell Signaling, 2125), anti-TNFα (AbCam, ab9739), anti-Vinculin (Santa Cruz, sc-73614). Following overnight incubation, the membrane was washed five times with TBST and incubated for 1 h at room temperature with horseradish peroxidase-conjugated secondary antibodies (Biorad) diluted in TBST containing 2% skim milk. The membrane was washed five times in TBST and proteins were detected using a chemiluminescence reagent (Lumi-light, Roche).

### Dual immunohistochemistry and immunofluorescence

Human surgical tumor tissue specimens were obtained from glioma patients under an institutional Research Ethics Board-approved protocol. The written informed consent was obtained from each patient. Mouse or human tissues were fixed in formalin and paraffin embedded. 5 µm formalin-fixed paraffin-embedded (FFPE) tissue sections were de-paraffinized in xylene and re-hydrated through descending alcohols. Heat-mediated antigen retrieval was performed with a high pH Tris-based antigen unmasking solution (H-3301, Vector laboratories, Burlingame, CA) and heated in a pressure cooker for 20 min. Sections were blocked with 5% bovine serum albumin (BSA) in PBST (1x PBS, 0.1% Triton X-100) for 1 h at room temperature. Primary antibody was diluted in PBST + 5% BSA and incubated overnight at 4 °C. Primary antibodies used were: anti-CD31 (AbCam, ab28364), anti-VCAM1 (Santa Cruz, sc-13160), anti-TNFα (AbCam, ab9739), anti-CD68 (DAKO, M0514) and anti-Integrin alpha 4/CD49D antibody (AbCam, ab202969). For immunohistochemistry, sections were washed with PBS and ImmPRESS alkaline phosphatase secondary reagent (Vector laboratories) was added for 1 h at room temperature and stained with Vector Blue (Vector Laboratories). Sections were then washed with PBS and the above procedure was repeated from the protein blocking step. The second primary antibody was developed with Vector Red (Vector Laboratories). For double staining with anti-CD31 and anti-VCAM1, sections were counterstained with methyl green (Vector laboratories). All sections were dehydrated in ethanol, cleared with HistoClear (National Diagnostics) and mounted with VectaMount (Vector Laboratories). For flow cytometry analysis to determine the expression of alpha 4 integrin on Raw264.7 cells and U973 cells as a positive control. Anti-Integrin alpha 4/CD49D antibody (ab202969) was diluted in 1:100 for both IHC and flow cytometry. Alexa 488 donkey anti-rabbit secondary antibody (A32790) was diluted in 1:1000.

### Multi-analyte inflammatory ELISA array

RAW264.7 cells were seeded in a 24-well plate. Cells were washed once with 1x PBS and incubated with GAM-CM for 24 h. Media was then harvested, centrifuged at 200xg for 5 min, and supernatant was analyzed using a Multi-Analyte ELISArray kit (Qiagen), according to the manufacturer’s instructions.

### Multi-analyte human cytokine array

NHA, U87 and U251 cells were cultured for 48 h in DMEM + 0.5% BSA. Cells were washed once with 1x PBS and medium was collected, centrifuged at 200xg for 5 min, and supernatant was analyzed using the Proteome Profiler Human Cytokine Array Kit (R&D). Imaging was conducted using the ChemiDoc MP Imaging System (Bio-Rad). Densitometry was performed with Image Lab 6.0.1 (Bio-Rad) to determine the relative expression of cytokines in the media of U87 and U251 cell lines compared to NHA from the multi-analyte human cytokine array.

### TNFα ELISA assay of GAM-CM and tumor tissue

RAW264.7 cells were seeded in a 96-well plate. Cells were washed once with 1 × PBS and incubated for 24 h in GAM-CM as described above or in DMEM + 0.5% BSA containing cytokines IL-8 or MCP-1 (CCL2) (Prospec Protein Specialists) at concentrations of 1, 10 and 50 nM. Then the cells were washed once with 1 × PBS and incubated in DMEM + 0.5% BSA for 24 h. The supernatant was collected and analyzed with the TNFα ELISA kit according to manufacturer’s instructions (Invitrogen). For the GL261 and U87 mouse models, tumor tissue and the contralateral normal brain tissue (non-tumor cells injected hemispheres) was collected as described below and analyzed with the TNFα ELISA kit. The optical density at 450 nm was measured with the SpectraMax 190 Microplate Reader (Molecular Devices). All experiments were repeated in triplicate. Values were relative to NHA after normalization and expressed as mean ± max/min (95% confidence interval).

### Neutralization or inhibition of TNFα

GAM-CM was prepared as above and incubated with 0.4 µg/mL anti-TNFα (AbCam) or MP6-XT22 (BioLegend) at room temperature with rocking for 1 h to allow for antibody binding. HUVEC or hCMEC/D3 cells were washed once with 1x PBS and pre-incubated medium or medium containing Thalidomide (Tocris) at a concentration of 10 µM was added. Cells were incubated for 24 h at 37 °C and subsequently analyzed by angiogenesis RT-PCR array.

### Analysis of BBB-permeability of MP6-XT22

Tumor-bearing, MP6-XT22 treated mice or controls were anaesthetized using 0.5 mg/g of intraperitoneal injection of Avertin (Sigma-Aldrich) and transcardially perfused with PBS. Tumor tissue and normal contralateral brain were excised, minced in cold Hank’s Balanced Salt Solution (HBSS) and homogenized on ice with a hand-held tissue disruptor. Homogenates were spun at 1000 × *g* for 10 min at 4 °C and pellet was resuspended in 1 mL 18% Dextran/HBSS. Samples were then centrifuged at 5400xg for 15 min. The supernatant (Brain-rich fraction) was collected (reserving pellet), transferred to a new tube with 1 mL 1% bovine serum albumin (BSA)/HBSS, centrifuged at 1500xg for 10 min, and resulting pellet was resuspended in 200uL lysis buffer (100 mM Tris, pH 7.4, 150 mM NaCl, 1 mM EGTA, 1 mM EDTA, 1% Triton X-100, 0.5% sodium deoxycholate). The pellet (capillary fraction) reserved from above, was washed 1 × with 1 mL 1% BSA/HBSS, and resuspended in lysis buffer. Samples were sonicated, centrifuged at 14,000 rpm for 10 min and analyzed with a Rat IgG ELISA (Invitrogen).

### GL261 and U87 mouse model

All animal procedures were carried out according to animal user protocols approved ethically by the Institutional Animal Care Committee under the guidelines of the Canadian Council on Animal Care and the University Health Network Research Ethics Board. The GL261 syngeneic mouse model was generated as follows: 2 × 10^5^ GL261 cells were suspended in 4 µL PBS and intracranially injected into the right frontal cortex of 6–8 weeks male and female C57BL/6 mice ( n = 20) as previously described [6]. One-week post-injection, mice were randomized into control or MP6-XT22 treatment groups (n = 10 for each group). Mice were treated every 3 days with intraperitoneal injections of 20 mg/kg MP6-XT22 until endpoint. The human GBM mouse xenograft model was generated as follows: 6–8 week of male and female NOD/SCID mice (n = 20) were subjected to 3 Gy total body irradiation (TBI) and reconstituted with RFP-BM cells. Ten reconstituted mice were intracranial injected with 2 × 10^5^ GFP-U87 cells to generate intracranial gliomas. Ten mice from both tumor bearing and non-tumor group were treated with B20.4.1.1 or vehicle control (n = 5 for each group). B20.4.1.1 (5 mg/kg) or vehicle control was administrated three times per week beginning one-week post- intracranial injection. Two -weeks post-treatments, we harvested tumor tissues, saved small piece for TNFα Elisa and morphological studies, IF and quantitative analyses for BMDC and aMVD from each tumor, processed the rest of tumor tissues and pooled all the cells for FACs. BM-derived macrophages (RFP+/F4/80+ cells) were sorted and pooled for RNA extraction and microarray analysis.

### Microarray data analysis

Microarray was performed on the Affymetrix GeneChip Scanner 3000 and the data were normalized with log2 transformation. Significantly upregulated genes (> twofold up) were selected for pathway analysis. A functional annotation analysis based on Database for Annotation, Visualization and Integrated Discovery (DAVID) was used to identify significantly enriched pathway. The heatmap was generated based on the fold changes of genes associated with the pathway.

### Human GBM glioma immunohistochemical analysis

Two cohorts of gliomas were selected for this study. One with 10 LGG samples (Grade II and III gliomas) and 14 IDH-wt GBM. We chose IDH-wt only for this preliminary analysis to have a uniform population by eliminating confounding results with IDH-mutant tumors. Two independent neuropathologists (KA and SK) scored the sections for extent of VCAM1 and CD31 staining. We correlated expression of VCAM1 and tumor grades. The second cohort included a total of 39 IDH-wt GBMs with 13 patients treated with Bevacizumab and 26 untreated patients, samples were obtained prior to initiation of treatment. The serial sections from each patient were co-immunostained with CD68 and TNFα or TNFα with VCAM1. Two independent neuropathologists (KA and SK) scored the sections for extent of VCAM1 staining. Digital analysis was performed on the HALO software version 2.1 (Indica Labs) using the random forest tissue classifier and multiplex immunohistochemistry module to identify positive TNFα and CD68 cells in tumor sections. The percentage of TNFα + and CD68+ cells/mm^2^ of tumor section was correlated to VCAM1 expression as well as OS following treatment with Bevacizumab.

### Survival and multivariate analysis

The gene expressions of 593 diffused gliomas were measured using the Illumina Hiseq RNASeqV2 and log2 transformed by the UCSC Cancer Browser team. The whole dataset was downloaded from the Genomic Data Commons (GDC) data portal (https://gdc.nci.nih.gov/). The diffuse gliomas consisting of 447 LGGs with 362 IDH-mut and 85 IDH-wt tumors and 146 GBMs with 9 IDH-mut and 137 IDH-wt tumors were selected for study since their corresponding patient survival data are available. We generated a boxplot to investigate the gene expression of VCAM1 among IDH-wt LGG and IDH-wt GBM. The Wilcoxon rank-sum test (two-sided) was performed to compare the two groups and calculate p values. Survival curve was produced by Kaplan–Meier method using survival package in R Bioconductor package and a log-rank test was performed to calculate p values where 0.05 is considered to be significant. Multivariate Cox analysis adjusted for confounding factors like grade and IDH status was performed to identify if VCAM1 gene expression is a significant predictor of survival independent of these confounding factors. Multivariate Cox analysis estimates the Hazard Ratio (HR), where HR > 1 is significantly associated with shorter survival with 95% Confidence Interval (CI).

### Statistical analysis

All in vitro experiments were performed in triplicate unless specifically stated in figure legends, with mean and s.e.m. reported. Unpaired Welch’s T-tests were used to compare the means of two experimental groups. Analysis of variance (ANOVA) was conducted for multi-group comparisons followed by a post-hoc Dunnett’s multiple comparison test to identify differences among experimental subgroups.

## Results

### GAMs induce EC activation

**Fig. 1:**
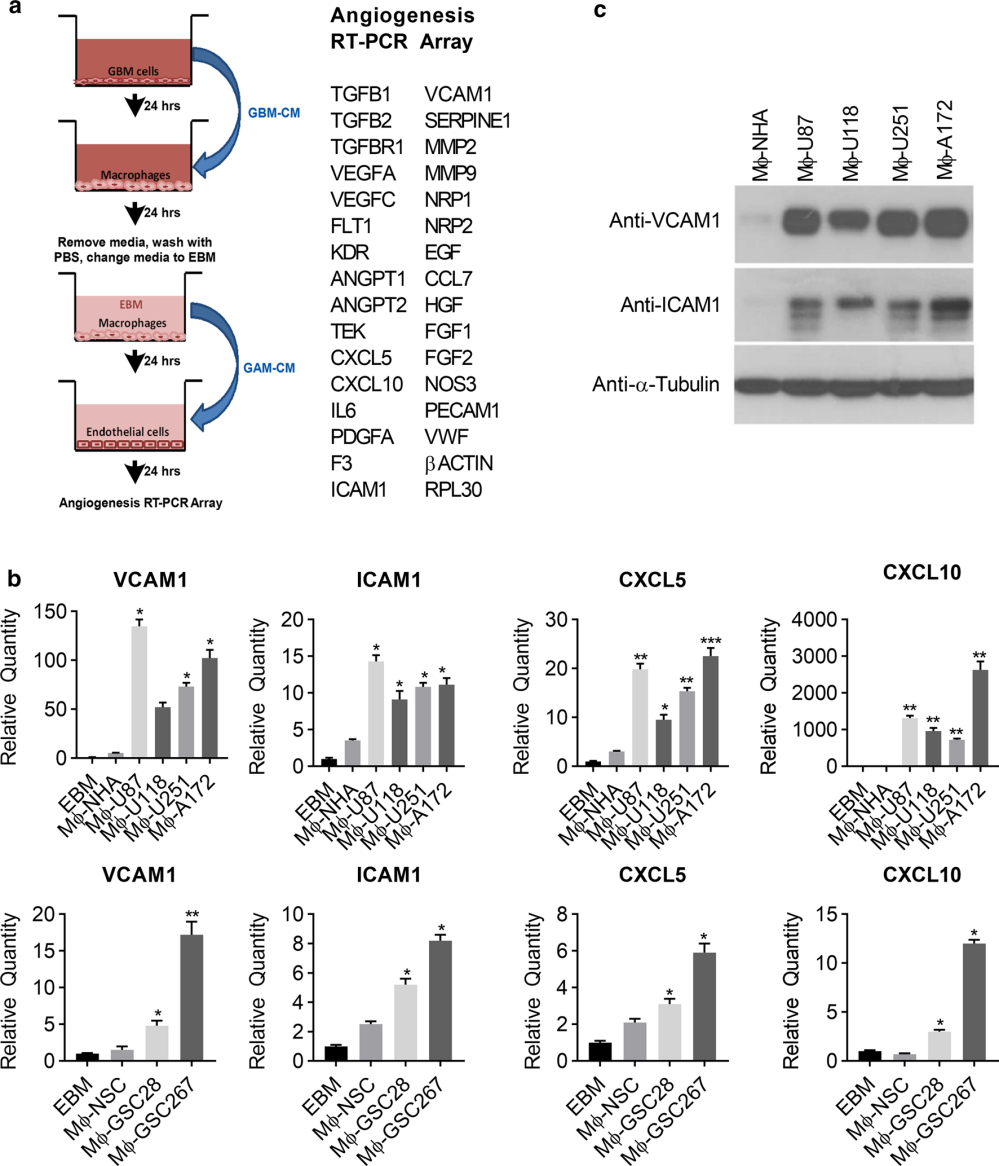
Glioma-associated macrophages induce EC activation. **a** Schematic representation of experimental design. **b** Human umbilical vein cells (HUVEC) were incubated with media alone (EBM) or conditioned medium (CM) from RAW264.7 macrophages stimulated with normal human astrocytes (Mφ-NHA) or GBM cell lines U87, U118, U251, A172 (Mφ-U87, Mφ-U118, Mφ-U251, Mφ-A172) (upper panel) and normal human neural stem cells (Mφ-NSC) or glioma stem cell lines GSC28, GSC267 (Mφ-GSC28, Mφ-GSC267) (lower panel). mRNA was extracted and analyzed by an RT-PCR angiogenesis array. Results were normalized to *RPL30* and *ACTB*. The relative quantity (RQ) of significantly altered genes (> twofold relative to Mφ-NHA or Mφ-NSC) are shown. n = 3, mean ± max/min, 95% confidence interval. *p < 0.05, **p < 0.01, ***p < 0.001 C. HUVEC were treated as described in (b), lysed, and immunoblotted with the indicated antibodies

To study the relationships between GAMs and EC activation, we first examined the effect of GAMs on the gene expression profile of ECs. To accomplish this, we designed and developed an in vitro model examining the differential expression of angiogenic genes involved in GBM vascularity (Fig. 1a, left panel). To model GAMs, we first stimulated macrophages (RAW 264.7 cells) with conditioned medium (CM) collected from GBM cell lines, selecting from a panel of GBM cells (U87, U118, U251, or A172) (Fig. 1b, upper panel) and glioma stem cells (GSC28 and GSC267) derived from primary human GBM patients (Fig. 1b, lower panel). Subsequently, CM from stimulated macrophages was used to stimulate ECs for 24 h. ECs gene expression was analyzed using an RT-PCR angiogenesis panel comprised of 30 angiogenesis genes (Fig. 1a, right panel). Treatment of ECs with CM from GAMs resulted in a significant upregulation (> twofold) of four of the thirty angiogenic genes: *VCAM1, ICAM1, CXCL5,* and *CXCL10* compared to control (Fig. 1b). Interestingly, VEGFA, a key angiogenic regulator was not significantly upregulated (> twofold) in Mφ-GBM cell lines when compared to Mφ-NHA, suggesting that GAMs induce EC activation independent of VEGFA (Additional file 1: Figure s1). Immunoblot analysis confirmed the increased expression of VCAM1 and ICAM1 proteins in ECs stimulated by GAM CM, validating the RT-PCR findings (Fig. 1c). Importantly, other tumor-stimulated (786-O, H1299, Hei193 and U2OS) macrophages, failed to induce expression of *VCAM1, ICAM1, CXCL5 and CXCL10* as shown in Additional file 1: Figures S2. Furthermore, human cerebral ECs (hCMEC/D3) respond similar to HUVECs when stimulated with GAM-CM (Mφ-U87) (Additional file 1: Figure S3). These results demonstrate that GAMs specifically induce EC activation.

### ECs activation associates with patient glioma grade and worse OS

**Fig. 2:**
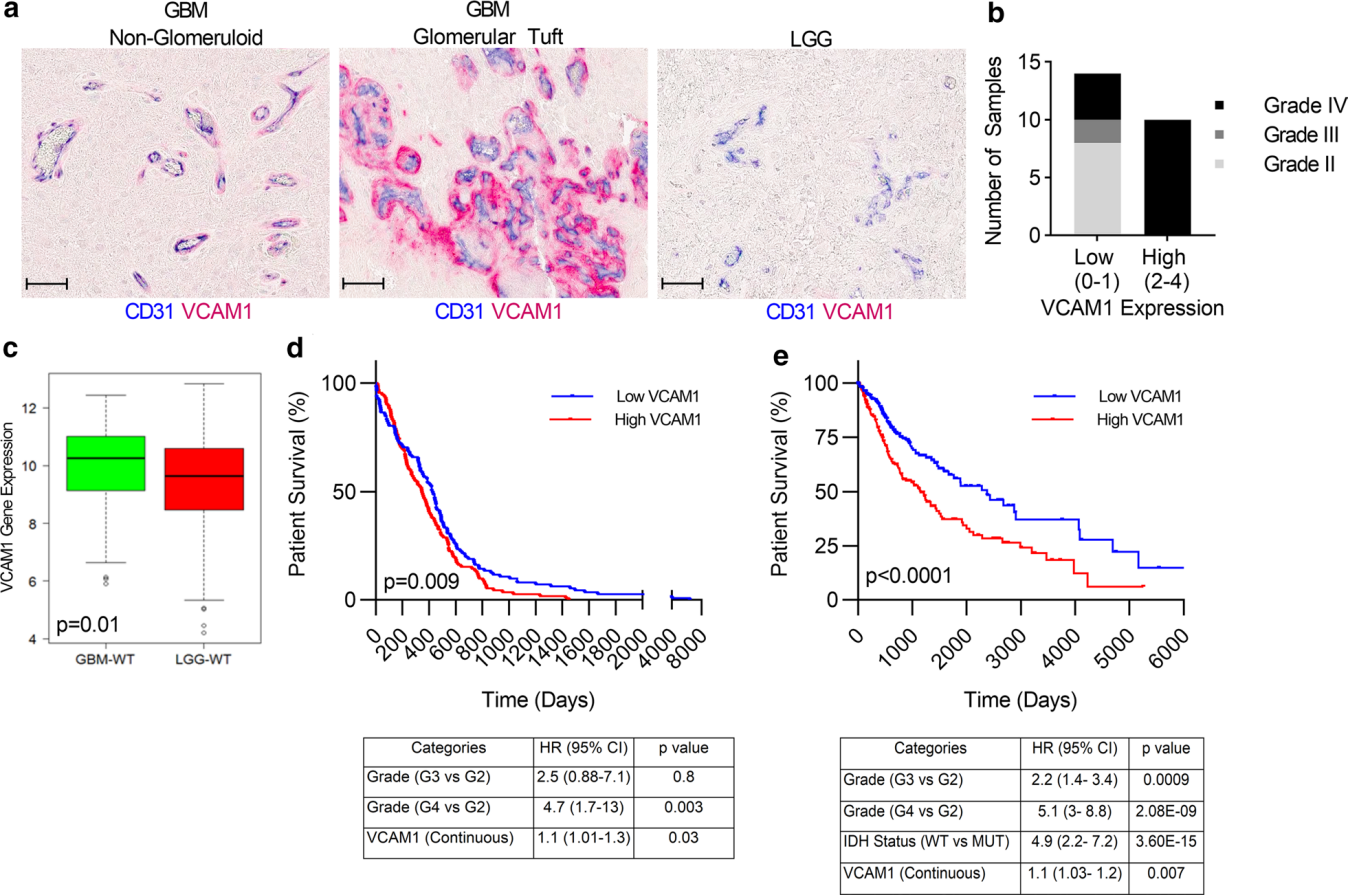
EC activation associates with patient glioma grade and worse overall survival. **a** Representative images from glioblastoma (GBM) or low-grade glioma (LGG) tissue samples co-stained with CD31 (blue) and VCAM1 (red). Classical “glomerular-tufts” seen in GBM showed a distinct VCAM1 positive stain (red) restricted to the “glomerular tufts” as seen with EC piling (CD31+, blue) around the lumen of a vessel. Non-glomerular vasculatures in the GBM were predominantly CD31 positive (blue). **b** Sections were scored as low (0–1) or high intensity (2–4) VCAM1 staining and the bar graph summarizes the number of samples from low grade gliomas (grade II or III) or high-grade glioma (IV) with low or high expression of VCAM1. (n = 24). Scale bar = 50 µm. **c** Mean *VCAM1* expression was calculated in 137 GBM IDH-wt and 85 LGG IDH-wt tumors, *p* = 0.01. **d** Kaplan–Meier survival curve of 222 IDH-wt gliomas grouped by high or low *VCAM1* expression (log-rank test, *p* = 0.009, upper panel). Multivariate analysis adjusted for confounding factors such as grade showed that *VCAM1* is a prognostic factor associated with survival (*p* = 0.03, lower panel). **e** Kaplan–Meier survival curve of 593 gliomas grouped by high or low *VCAM1* expression (log-rank test, *p* < 0.0001, upper panel), multivariate analysis adjusted for confounding factors such as tumor grade and IDH status showed that VCAM1 is a prognostic factor associated with survival (*p* = 0.007, lower panel)

ECs activation is characterized by increased expression of surface adhesion molecules such as VCAM1 and ICAM1 [13] and there is evidence that VCAM-1 is closely associated with tumor neoangiogenesis and progression [20]. To investigate the correlation of VCAM1 expression and glioma grades, we analyzed a cohort of IDH-wildtype(wt) human grade IV (GBM) (n = 14) and lower grade (LGG, grade II and III) glioma samples (n = 10) using dual immunohistochemical (IHC) staining for the endothelial marker CD31, and endothelial activation marker VCAM1. The classical glomerular-tufts seen in GBM, which are histological structures formed as a consequence of ECs proliferation and piling, showed a distinct VCAM1 positive staining restricted to the EC tufting and seen around the lumen of a vessel. Non-glomerular vasculature in the GBM by comparison were predominantly CD31 positive with low VCAM1 expression (Fig. 2a). We found significantly higher expression of VCAM1 in GBM cases (10/14) compared to LGG, where there was minimal to absent VCAM1 staining detected in all 10 cases examined (Fig. 2a, b).

To further validate these findings, we analyzed the TCGA glioma RNAseq database downloaded from the Genomic Data Commons (GDC) data portal (https://gdc.nci.nih.gov/) and analyzed VCAM1 expression in 137 IDH-wt GBM and 85 IDH-wt LGG samples. *VCAM1* gene expression was statistically significantly higher in GBM vs. LGG (*p* = 0.01, Fig. 2c). Higher *VCAM1* expression was associated with worse outcome as determined by median survival at 347 days vs. 427 days with lower expression of VCAM1 (log-rank test, *p* = 0.009, n = 222, Fig. 2d, upper panel). Multivariate analysis was performed in the IDH-wt glioma cohort (n = 222) to define the prognostic factors that contribute to OS. We found *VCAM1* expression as a significant prognostic factor independent of tumor grade with HR > 1, indicating that it is significantly associated with shorter survival with 95% CI (*p* = 0.03, Fig. 2d, lower panel). We expanded our cohort to include all IDH-wt and IDH-mut gliomas in the TCGA RNAseq database (n = 593). Strikingly, *VCAM1* expression remained a predictor of OS with median survival at 1152 days in *VCAM1* high expression group vs. 2379 days in low expression group, which was based on median *VCAM1* expression cutoff (*p* < 0.0001, Fig. 2e, upper panel). *VCAM1* expression was significant in multivariate analysis, independent of tumor grade or IDH status with HR > 1, indicating that it is significantly associated with shorter survival with 95% CI (*p* = 0.007, Fig. 2e. lower panel). These results collectively support VCAM1 to be an important marker of activated ECs and that ECs activation as measured by high expression of VCAM1 can serve as a predictor of poor outcome in gliomas.

### TNFα secreted by GAMs induces ECs activation and associates with OS in human GBM

**Fig. 3:**
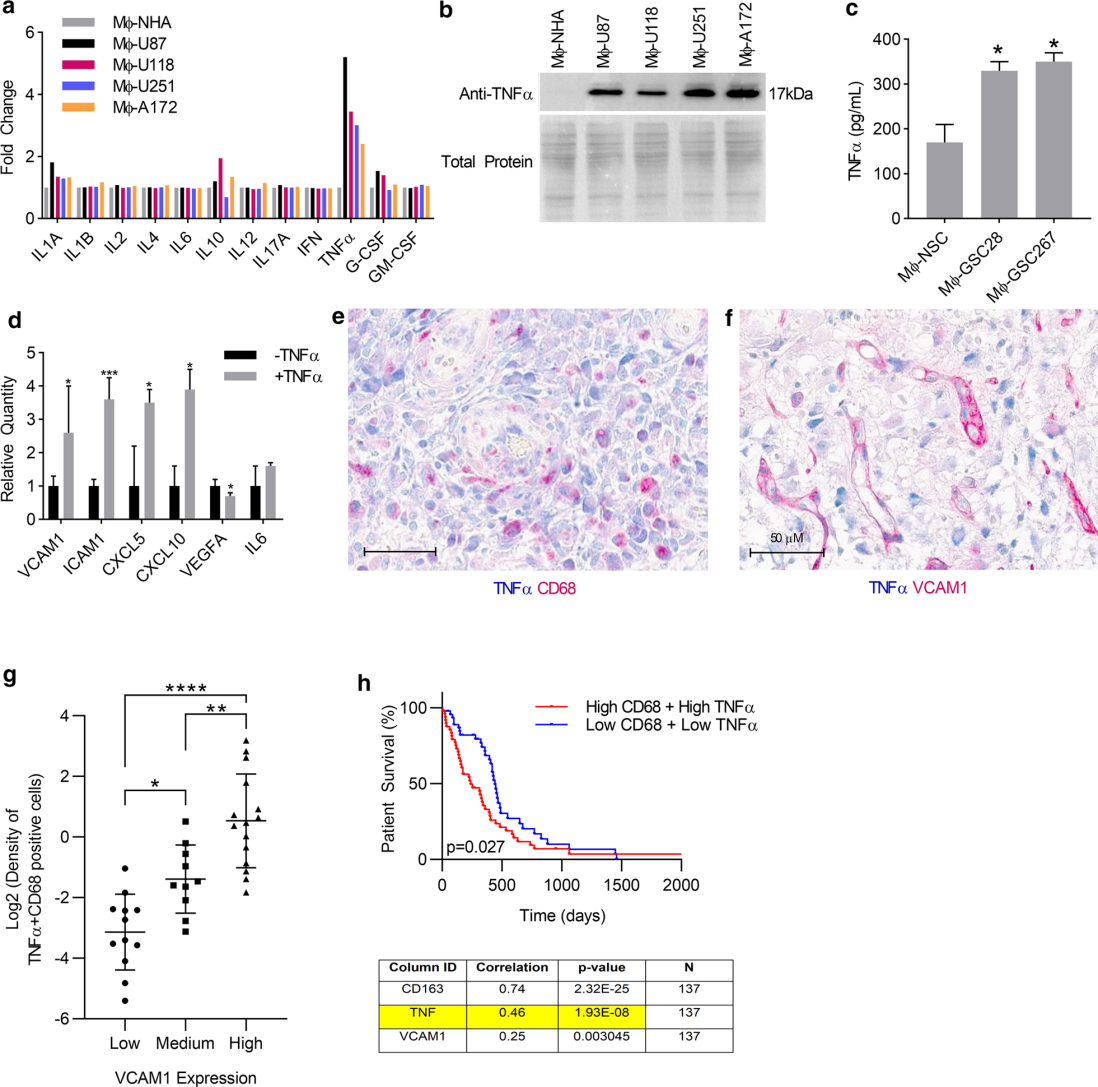
TNFα secreted by GAMs induces EC activation and associates with OS in human GBM. **a** CM from Mφ-NHA, Mφ-U87, Mφ-U118, Mφ-U251, or Mφ-A172 was analyzed by a multi-analyte inflammatory ELISA array or **b** concentrated and immunoblotted with anti-TNFα. Total protein was detected using PageBlue. **c** TNFα ELISA with CM from Mφ-NSC, Mφ-GSC28 or Mφ-GSC267 was performed to validate the results found in the GBM cell lines. **d** Human cerebral endothelial cells (hCMEC/D3) were incubated with or without 10 ng/mL hTNFα for 24 h and gene expression was analyzed on an RT-PCR angiogenesis array. Relative quantity was calculated by normalizing to –TNFα. n = 3, mean ± max/min, 95% confidence interval *p < 0.05, **p < 0.01, ***p < 0.001. **e** Representative image of human GBM tissue section co-stained with TNFα (blue) and CD68 (red), double positive cells (arrow). **f** Representative image of human GBM tissue section co-stained with TNFα (blue) and VCAM1 (red). Scale bar = 50 µm. g. Correlation between extent of VCAM1 staining (Low, Medium or High) and percentage of TNF-alpha expressing GAMs cells per mm2 tumor area was performed in 39 IDH-wt GBM tissues. Log2 transformation was performed on the values of percentage of TNF-alpha expressing GAMs cells per mm2 tumor area (R = 0.8, *p* = 2.738e-009). h. Correlation of expression between CD68 and TNFα was analyzed in 137 IDH-wt GBM, R = 0.46, *p* = 1.93E-08 (lower panel), Kaplan–Meier survival curve of 137 IDH-wt GBM grouped by high or low CD68 and TNFα expression, *p* = 0.027 (upper panel)

We next wanted to uncover the specific cytokines secreted by GAMs that induce EC activation. We performed a multi-analytic inflammatory ELISA on conditioned medium from macrophages that were stimulated with the same panel of GBM cell lines (Mφ-GBM) as outlined above compared to macrophages conditioned with normal human astrocyte media (Mφ-NHA), which served as a control. Of the twelve inflammatory cytokines analyzed, only TNFα was significantly upregulated more than two-fold in all media obtained from Mφ-GBM (*p* < 0.05, Fig. 3a). This was further supported by Western immunoblot analysis showing that Mφ-GBM contains higher levels of TNFα in comparison to Mφ-NHA (Fig. 3b). Additionally, we show that TNFα expression is higher in the media from two primary glioma stem cell lines Mφ-GSC28 and Mφ-GSC267 compared to neural stem cell (NSC) control (Mφ-NSC, Fig. 3c).

Next to validate the effects of TNFα on EC activation, we treated human cerebral endothelial cells (hCMEC/D3) with recombinant human TNFα and found that it induced a statistically significant upregulation of *VCAM1*, *ICAM1*, *CXCL5*, and *CXCL10 and* no upregulation of *VEGFA*, similar to the effects seen with Mφ-GBM (Figs. 3d, 1b, Additional file 1: Figure s1). Taking together, our data strongly support that TNFα is the cytokine secreted by GAMs that induces EC activation.

To examine clinical significance of our findings, we investigated if human GAMs express TNFα and whether this associates with EC activation and OS. We used one of the cell surface macrophage markers, CD68 to identify GAMs [39]. CD68 is one of the most useful and descriptive markers for microglial function since both M1 polarized and M2 polarized microglia/macrophages can express CD68 [22]. We have also used F4/80 and MAC3 to confirm GAMs (data not shown). In a cohort of 39 human IDH-wt GBM patients, the serial sections from each patient were co-immunostained with CD68 and TNFα or TNFα andVCAM1. Dual IHC showed that GAMs (CD68+ cells) express TNFα at a range from 0 to 98.44% with average of 47.53% (Fig. 3E, Table 1). Co-staining for TNFα and VCAM1 in human GBM tissues also revealed a regional association of high VCAM1 and TNFα staining (Fig. 3F). Furthermore, we discovered that there is significantly positive correlation between extent of VCAM1 staining (Low, Medium or High) and percentage of TNF-alpha expressing GAMs cells per mm2 tumor area (Spearman, R = 0.8, *p* = 2.738e-009, Fig. 3g, Table 1).

Using publicly available RNAseq data from TCGA, correlation of expression between CD68 and TNFα was analyzed in 137 IDH-wt GBM. The expression of TNFα was significantly correlated with CD68 expression (Spearman, R = 0.46, *p* = 1.93E-08, Fig. 3h lower panel) and co-expression of TNFα and CD68 was significantly associated with worse OS (Spearman, *p* = 0.027, Fig. 3h upper panel). Additionally, this analysis showed that CD68 expression was significantly associated with another characteristic macrophage marker CD163 (Spearman, R = 0.74, *p* = 2.32E-25) and also with VCAM1 expression (Spearman, R = 0.25, *p* = 0.003) (Fig. 3h lower panel). Collectively, these results support that TNFα is positively correlated with the macrophage marker CD68 and worse OS in patients with GBMs.

### GBM cells secrete interleukin 8 (IL8) and chemokine (C–C motif) ligand 2 (CCL2) to induce TNFα secretion in GAMs

**Fig. 4:**
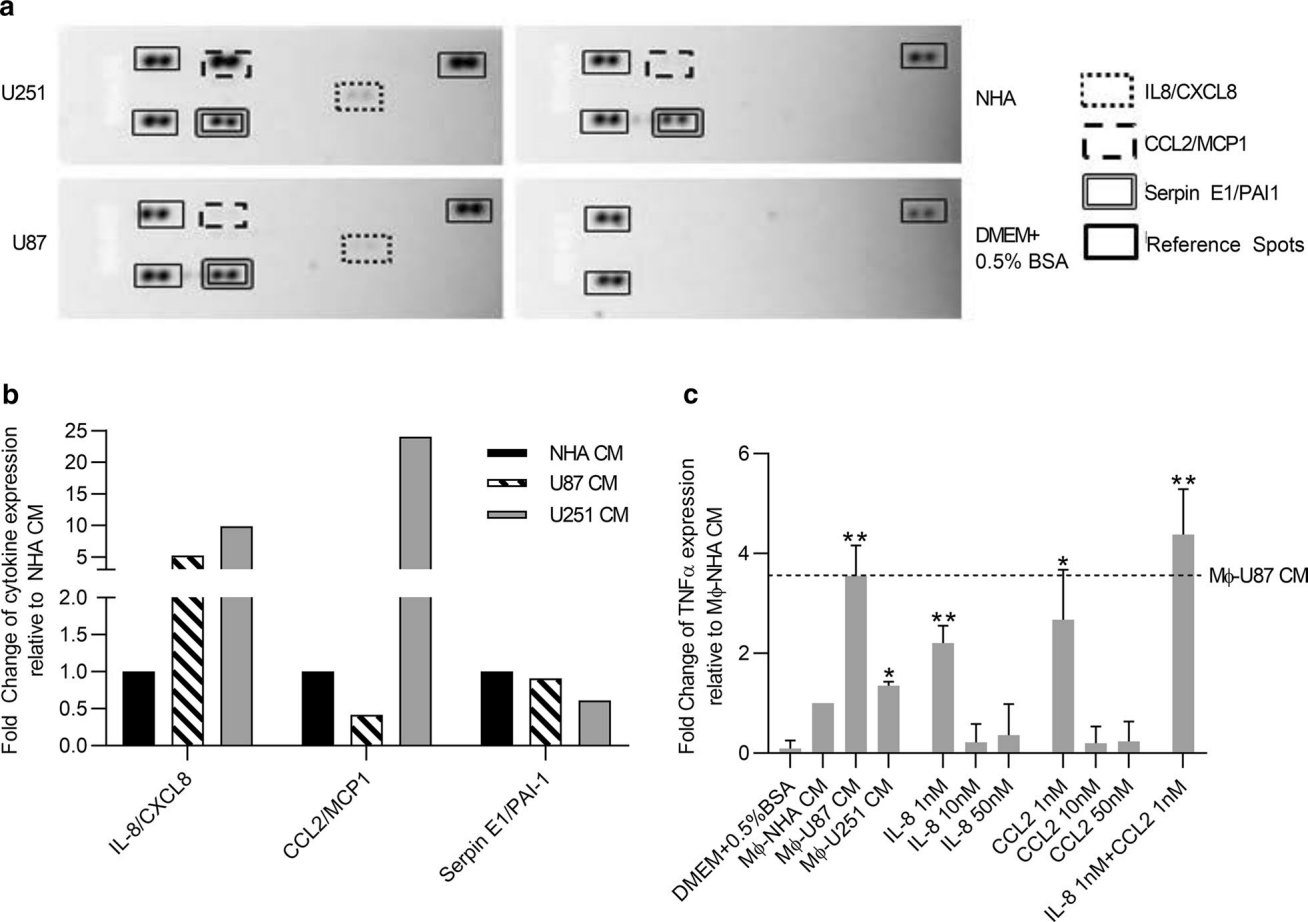
Glioma-secreted IL8 and CCL2 regulate GAMs to secrete TNFα **a** CM from NHA, or glioma cell lines was analyzed by a Proteome Profiler Human Cytokine Array and **b** densitometry was performed to determine the relative expression of cytokines in the CM of glioma cell lines compared to NHA. **c** RAW264.7 macrophages were treated with CM from NHA, U87, U251 or DMEM + 0.5% BSA containing cytokines IL-8 and/or CCL2 at concentrations of 1, 10 and 50 nM. After 24 h incubation, cells were washed once with 1x PBS and cultured in fresh DMEM + 0.5%BSA for 24 h. The supernatant was then collected and secreted TNFα was detected with TNFα ELISA kit. The relative expression of TNFα was normalized to NHA. n = 3, mean ± max/min, 95% confidence interval. *p < 0.05, **p < 0.01

To determine the factors secreted by GBM cells that in turn regulate GAMs to produce TNFα, we conducted a cytokine array analyzing 24 cytokines. Specifically, CM from different human GBM cell lines and NHA controls were collected and hybridized on a human cytokine array (Fig. 4a). We found that IL-8/CXCL8 secretion was increased in both U87 and U251 CM by 5.2- and 9.9-fold respectively compared to NHA CM. The secretions of CCL2/MCP-1 in U87 and U251 CM were 0.4 and 24-fold compared to NHA CM. Serpin E1/PAI-1 was also upregulated however since secreted by both GBM and NHA cell lines to similar levels (0.70–onefold, Fig. 4b) for subsequent studies, we focused on IL-8 and CCL2. To determine the key factor(s) and their optimal dosage responsible for GAM-induced TNFα secretion, we exposed RAW 264.7 macrophage cells to IL-8 or CCL2 alone or in combination at concentrations of 1 nM, 10 nM or 50 nM and compared the levels of TNFα secretion to that induced by Mφ-NHA CM (negative control), Mφ-U87 CM and Mφ-U251 CM (two positive controls). Consistent with our previous findings, we found that both Mφ-U87 CM and Mφ-U251 CM significantly induced TNFα secretion compared to Mφ-NHA CM (*p* < 0.05, Fig. 4c). Secondly, Mφ-U251 CM induced significantly less secretion of TNFα than Mφ-U87 CM even though U251 CM contains relatively higher amounts of IL-8 and CCL2 than U87 CM (9.9- vs. 5.2-fold and 24- vs. 0.4-fold, respectively, Fig. 4b, c), which suggests a non-linear relationship between either IL-8 or CCL2 and TNFα. Indeed, we establish this to be true since exposure of RAW264.7 cells to IL-8 or CCL2 alone or in combination at concentrations of 1 nM shows higher stimulation of TNFα secretion compared to those at concentrations of 10 or 50 nM (Fig. 4c). Thirdly, we found that while IL-8 or CCL2 alone significantly stimulates GAMs to secrete TNFα compared to NHA CM, only the combination of IL-8 and CCL2 stimulates the secretion of TNFα to the level comparable to U87 CM, which is the highest degree of response (Fig. 4c). Taken together, we found that both IL-8 and CCL2 are required to stimulate TNFα secretion in GAMs.

### Inhibition of TNFα prevents EC activation and prolongs survival of mouse glioma model

**Fig. 5:**
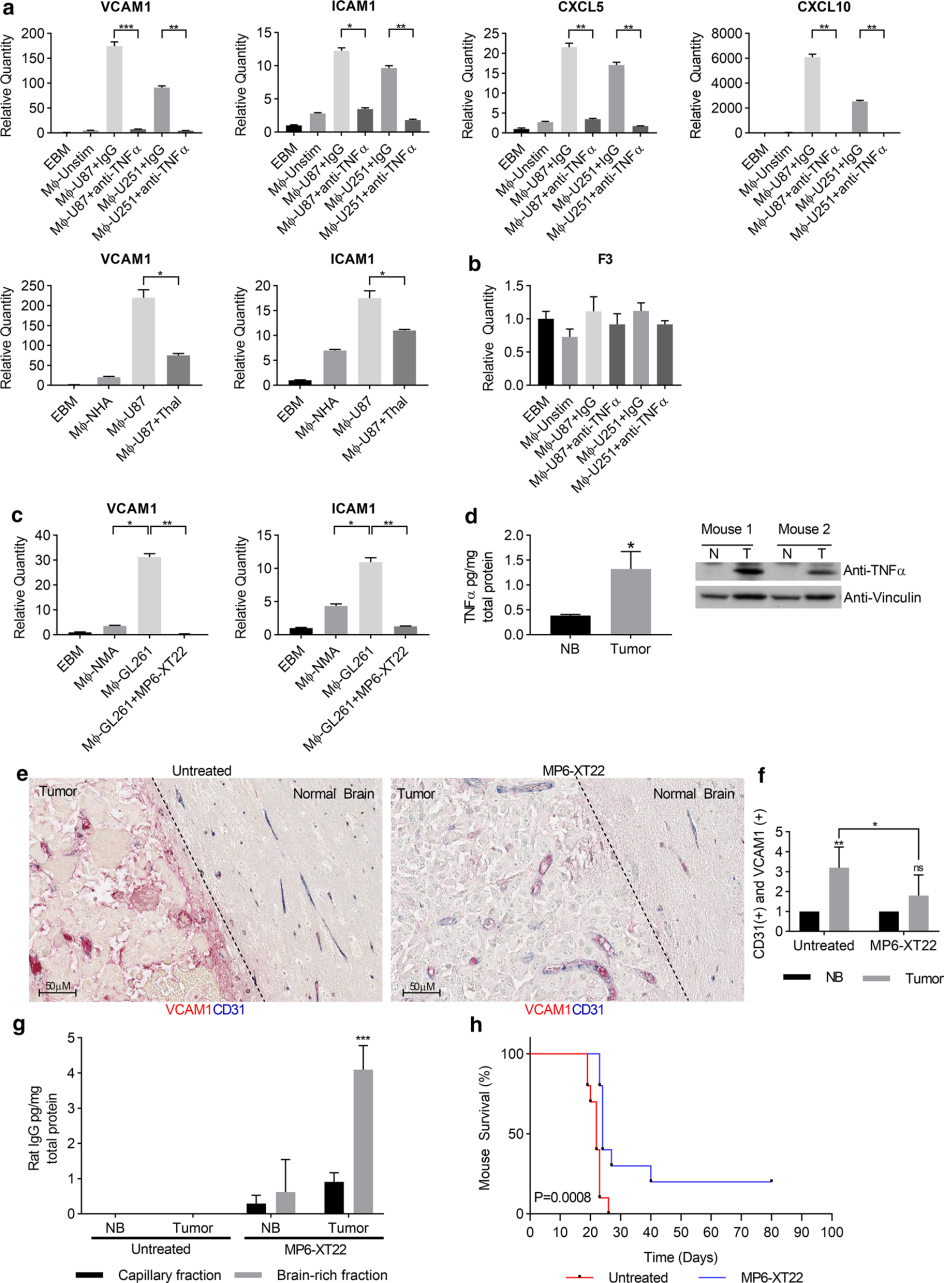
Inhibition of TNFα prevents EC activation and prolongs survival of mouse glioma model **a** HUVECs were incubated with EBM or CM from Mφ-U87 or Mφ-U251 in the presence or absence of 0.4 µg/mL anti-TNFα neutralizing antibody or IgG (upper panel) or thalidomide at concentration of 10 µM (lower panel). Relative quantities of genes were analyzed by RT-PCR and normalized to EBM. n = 3, mean ± max/min, 95% confidence interval. **b** HUVEC were incubated with EBM media alone (Mφ-Unstim) or CM from RAW264.7 cells stimulated with glioma cell lines (Mφ-U87 or Mφ-U251) in the presence or absence of 0.4 µg/mL anti-TNFα neutralizing antibody or IgG. Relative quantities of F3 were analyzed by RT-PCR and normalized to EBM. n = 3, mean ± max/min, 95% confidence interval. **c** hCMEC/D3 were incubated with CM from RAW264.7 macrophages stimulated with normal mouse astrocytes (Mφ-NMA) or GL261(Mφ-GL261) cells in the presence or absence of MP6-XT22. Gene expression was analyzed as in (**a**). **d** Protein expression of TNFα in tumor (T) versus normal contralateral brain tissue (NB) isolated from GL261 syngeneic mice was analyzed by ELISA, mean ± SD, n = 3 or immunoblotting. **e** Representative images from GL261 syngeneic mice brain tissue co-stained with CD31 (blue) and VCAM1 (red). GL261 tumor-bearing mice were treated with vehicle control (left) or MP6-XT22 (right) (Scale bar = 50 µm). **f** Co-expression of CD31 and VCAM1was scored by two independent pathologists with adjacent normal brain area or normal contralateral tissue (NB) scoring as 1 (n = 10). **g** Capillary-depleted brain rich fractions and capillary fractions from untreated and MP6-XT22 treated mice were isolated from NB and tumor tissue, lysed, and analyzed with a rat IgG ELISA. Concentrations were normalized to total protein, mean ± SD, n = 5. **h** Kaplan–Meier survival curve of GL261 syngeneic mice randomized into control (n = 10) or MP6-XT22-treated (n = 10) groups. *p* = 0.0008. *p < 0.05, **p < 0.01, ***p < 0.001

To examine whether inhibition of GAM-secreted TNFα reduces EC activation, we inhibited TNFα with a neutralizing antibody or the selective inhibitor, Thalidomide, and evaluated the effects on EC activation genes signatures [32]. We showed that inhibition of TNFα with a neutralizing antibody was sufficient to block GAM-induced upregulation of *VCAM1, ICAM1, CXCL5,* and *CXCL10* (Fig. 5a, upper panel), but had no effect on the expression of *coagulation factor III (F3)* that was not altered by GAM-CM (Fig. 5b). Inhibition of TNFα with Thalidomide also significantly downregulated the expression of *VCAM1* and *ICAM1,* the two main genes characterized for EC activation [13] (Fig. 5a, lower panel).

We next examined the therapeutic potential of targeting TNFα in the established syngeneic glioma mouse model, GL261. GL261 mouse model is an immunocompetent model that allows for the engraftment of tumors in the brain without immediate immune rejection. This model is typically used to investigate the role of the tumour microenvironment [18] and it is the most widely used syngeneic model of glioblastoma [27]. This model recapitulates many of the genetic and phenotypic characteristics of GBM, including mutations in p53 and K-Ras that drive high expression of c-myc, cellular pleomorphism, angiogenesis, and pseudopalisading necrosis [35, 40]. Although IL-8 and its major cognate receptor, CXCR1, are not present in mouse cells, MIP-2 and KC are suggested to be functional homologues [17, 23, 24, 43]. Both MIP-2 and CCL2 are expressed by GL261 tumor cells [5, 9, 42]. Consistent with human GBM cell lines, CM from GL261 stimulated macrophages (Mφ-GL261) which in turn upregulated expression of *VCAM1* and *ICAM1* but not VEGF compared to macrophages conditioned with normal mouse astrocytes (Mφ-NMA) (Fig. 5c, Additional file 1: Figure s4). Importantly we show that baseline expression of TNFα in GL261-injected hemisphere (T) is elevated relative to non-GL261 injected hemisphere (N) as determined by ELISA and Western blotting (Fig. 5d).

To investigate the impact of the increased levels of the TNF-α expression on EC activation in GL261 syngeneic glioma mouse model, we performed double IHC staining with CD31 and VCAM1 antibodies. We assess the co-expression of the CD31 and VCAM1 on endothelial cells in the tumor and adjacent morphologically normal cortical brain tissue in this mouse model. There is significantly increased co-expression of the CD31 and VCAM1 in tumor areas compared to that in adjacent normal brain tissue or non-injected contralateral normal brain (scores 3.2 vs 1, *p* = 0.0004, Left panels in Fig. 5e, f). These findings support that the GL261 glioma model recapitulates the increased TNFα expression that induces EC activation, as seen in human GBMs detailed in the results above.

To further investigate the significance of the TNFα inhibition on the GL261 syngeneic glioma mouse model tumorigenesis, we used MP6-XT22, the TNFα monoclonal antibody derived from rat, which is an analog of Infliximab, a chimeric monoclonal antibody directed at TNFα [25, 25]. We confirmed that MP6-XT22 inhibits EC activation induced in vitro by macrophages stimulated with GL261glioma cells, as shown in Fig. 5c. Subsequently, we examined the therapeutic potential of MP-XTT22 in vivo using the mouse GL261 glioma model. First, we show that MP6-XT22 can cross the blood–brain barrier (BBB) as detected by the presence of rat IgG in capillary-depleted brain rich fractions isolated from GL261 tumors in treated mice and was also found at significantly higher levels in tumor versus normal contralateral brain (non- GL261 injected hemisphere) by ELISA (Fig. 5g). Next, we treated GL261 tumor-bearing mice with vehicle control or MP6-XT22 to determine the therapeutic effect of MP6-XT22 on EC activation and OS. MP6-XT22 was shown to decrease co-expression of CD31 and VCAM1 in the tumor areas compared to those in untreated vehicle control (score 3.2 vs 1.8, *p* = 0.03, right panels in Fig. 5e, f) and increase survival (log-rank test, *p* = 0.0008, Fig. 5h). The treatment reduced the level of co-expression of VCAM and CD31 in tumor areas to that in normal brain tissues (score 1.8 vs 1, *p* = 0.06, right panels in Fig. 5e, f) which suggests normalized ECs.

Taken together, these data demonstrate that inhibition of TNFα reduces EC activation and prolongs survival of mouse glioma models and provides support for TNFα serving as a novel therapeutic target in GBM.

### Increased macrophage recruitment and local concentration of TNFα drive resistance to AATx

**Fig. 6:**
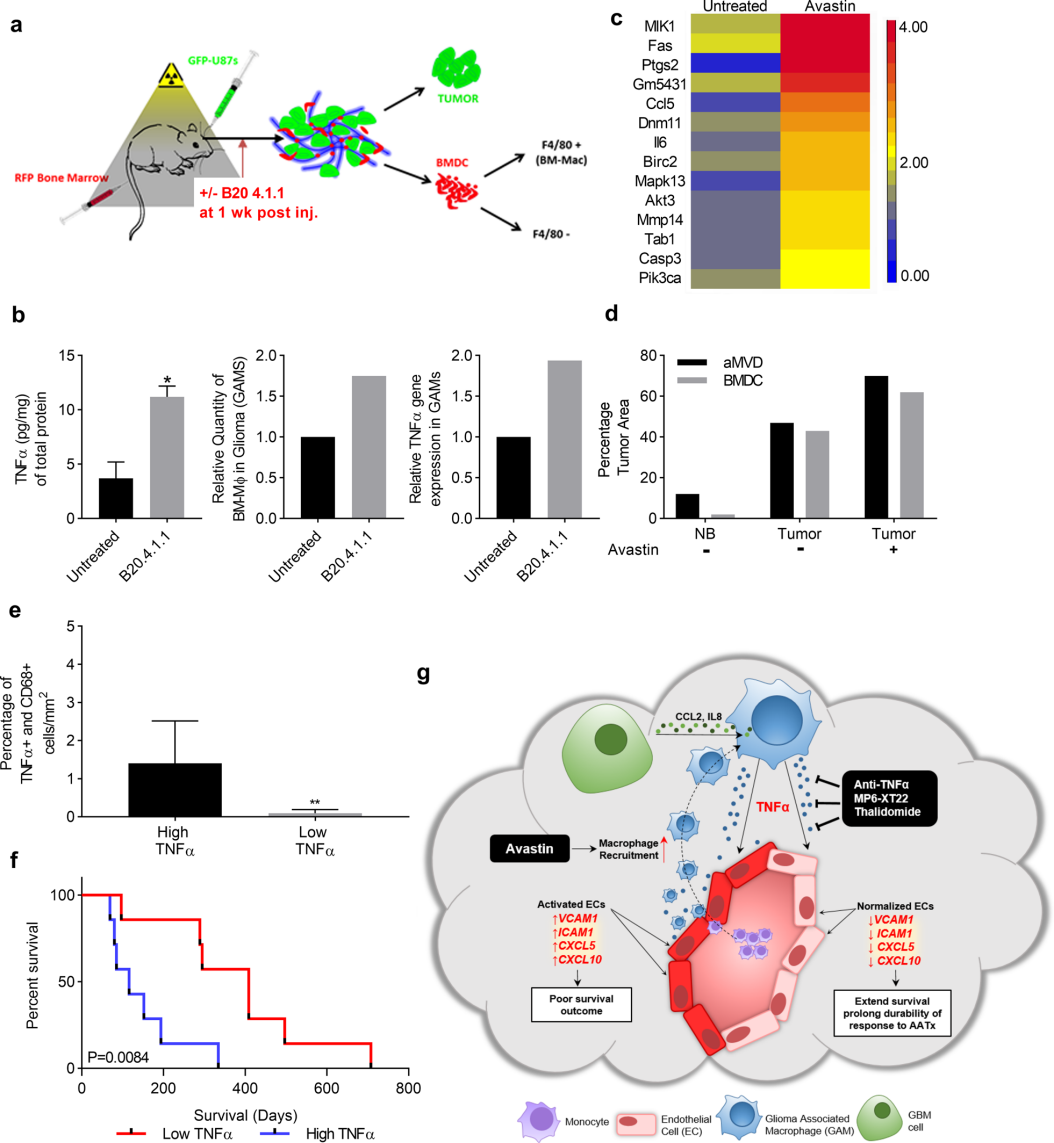
Increased macrophage recruitment and high local concentration of TNFα drives resistance and predicts failure of response to AATx **a** NOD/SCID mice with reconstituted RFP-Bone Marrow cells, used to generate intracranial gliomas with GFP-U87 cells (n = 20). Ten reconstituted mice were injected with GFP-U87 to generate intracranial gliomas. Ten mice from both tumor bearing and non-tumor group were treated with B20.4.1.1 treatments and vehicle controls one-week post intracranial injection (n = 5 for each). Tumors were harvested after two weeks of treatment, small portion from each tumor was saved for protein extraction and morphological studies, the rest of tumors were processed into single cell suspension and pooled prior to FACS. BM-derived macrophages (RFP+/F4/80+ cells) were sorted and pooled for RNA and protein extraction. **b** TNFα ELISA was performed on both untreated and B20.4.1.1 treated U87 xenograft tumor tissues (n = 5, left panel). Percentage of BM-derived macrophages (GAMs, RFP+/F4/80+ cells) in tumor cells (GFP cells) were analysed by flow cytometry (middle panel, pooled sample). TNFα gene expression in GAMs was determined by quantitative PCR (right panel, pooled sample). **c** Heatmap of fourteen genes associated with TNFα signaling pathway in untreated or B20.4.1.1 treated GAMs. **d** Double immunofluorescence staining was performed with CD31 and VCAM1 on the tumor sections. Percentage of bone marrow derived cells (BMDC, red fluorescence) and activated microvascular density (aMVD, CD31 and VCAM1 double positive cells) were determined by digital analysis in normal brain (NB) and tumors, with and without treatment of B20.4.1.1. **e** Thirteen human GBM samples collected prior to Bevacizumab treatment were stained by dual immunohistochemistry with anti-TNFα and anti-CD68. The percentage of double positive cells in CD68(+) cells per mm^2^ of tumor section was determined by digital analysis and two groups were divided based on median value. **f** Kaplan–Meier survival curve of high TNFα (n = 7) and low TNFα (n = 6) groups, *p* = 0.0212. **g** Graphic summary: GBM cells secrete IL-8 and CCL2 which stimulate GAMs to produce TNFα. Subsequently, TNFα induces a distinct gene expression signature of activated ECs including VCAM-1, ICAM-1, CXCL5, and CXCL10. High expression of VCAM1 correlates with worse survival outcome in IDH-wt glioma patients. Inhibition of TNFα with antibodies or drugs inhibits GAM-induced EC activation, improves survival and prolongs durability of response to AATx. *p < 0.05

It has been reported that in response to Bevacizumab, an inhibitor targeting VEGF, GBMs recur in a more aggressive manner and are highly invasive and resistant to additional treatment [10, 15]. The other study has also shown that anti-VEGF treatment increases hypoxia which promotes macrophage infiltration [14, 16, 26]. We posit that increased macrophage recruitment will increase the local concentration of TNFα which in turn promote EC activation. To understand the role of TNFα in glioma resistance to bevacizumab, a bone marrow (BM) chimera-human GBM xenograft mouse model was developed. The BM of NOD/SCID mice were reconstituted with red fluorescent protein (RFP)-BM cells to create a chimeric mouse and then GFP-U87 cells were injected intracranially to create an orthotopic GBM xenograft. B20.4.1.1, the mouse analog of Bevacizumab or vehicle control was administrated one-week post intracranial injection of cells and the tumors were harvested two-weeks after treatment. The tumors were processed into single cell suspension prior to fluorescence-activated cell sorting (FACS). Both U87 tumor cells (GFP+) and BM-derived macrophages (RFP+/F4/80+ cells) were sorted and pooled for RNA extraction and microarray analysis (Fig. 6a). We detected a significant increase in TNFα expression in the B20.4.1.1 treated GBM xenografts compared to control as shown by ELISA analysis (n = 5, *p* < 0.05, Fig. 6b, left panel). The percentage of BM-derived macrophages (RFP+/F4/80+ cells) in tumor cells (GFP cells) were analysed by flow cytometry. B20.4.1.1 treatment increased the amount of BM-derived macrophages in tumors (GAMs) by 1.9 fold compared to control (Fig. 6b, middle panel). Additionally, B20.4.1.1 upregulated TNFα gene expression in these GAMs as determined by quantitative PCR in RNA sample extracted from pooled cells sorted from 5 different tumors (Fig. 6b, right panel). Most strikingly, all fourteen genes in the TNFα signaling pathway were upregulated by greater than twofold in GAMs treated with B20.4.1.1 compared to control (Fig. 6c). The upregulation of TNFα in GAMs was associated with an increase in activated ECs (as measured by aMVD) in B20.4.1.1 treated tumors (Fig. 6d). These experimental data show that B20.4.1.1 treatment stimulates macrophage recruitment to GBMs and increases the local concentration of TNFα, which in turn promotes EC activation and may contribute to resistance to AATx.

### Upregulation of TNFα in GAMs predicted failed response to AATx

We sought to determine whether the above experimental findings were relevant to human GBM samples treated with Bevacizumab. To date, two phase III clinical trials with Bevacizumab have failed in human GBMs. Our study herein demonstrates that elevated TNFα expression in GAMs induced EC activation, providing a possible mechanism for resistance to AATx. To examine whether failure of AATx is due to elevated TNFα expression in GAMs, we examined expression of TNFα in CD68+ GAMs in GBM samples that were collected and processed prior to treatment with Bevacizumab and correlated the results with clinical outcome, using OS in Bevacizumab treatment as the end-point. We performed co-immunostaining of TNFα and CD68 on 13 GBM samples from patients to detect TNFα expression in GAMs per mm^2^ of tumor section. TNFα high and low groups were separated by median value (Fig. 6e) and correlated to survival following treatment with Bevacizumab. Patients in the low TNFα group had a median survival of 352 days following start of therapy, while patients in the high TNFα group had a median survival of 116 days after treatment with Bevacizumab, demonstrating that TNFα levels predict a statistically significant difference in response to Bevacizumab (log-rank test, *p* < 0.05, Fig. 6f). These results suggest that TNFα expression in GAMs can predict response to AATx with Bevacizumab in GBM patients and provides support for combining TNFα inhibition and Bevacizumab in clinical trials.

## Discussion

In this study, we report a new mechanism of endothelial cell activation in GBM, which is mediated by TNFα secreting GAMs. We show that GBM cells secrete two important cytokines, IL-8 and CCL2, which stimulate GAMs to produce TNFα. Secreted TNFα then activates ECs to express a gene signature indicative of EC activation, including increased expression of *VCAM1*, *ICAM1*, *CXCL5*, and *CXCL10* (Fig. 6g). In fact, we show that high expression of *VCAM1* correlates with worse survival outcome in IDH-wt glioma patients. Furthermore, we show that inhibition of TNFα with antibodies or drugs, inhibits GAM-induced EC activation and improves survival in a mouse glioma model. Importantly we show that high TNFα expression predicts worse response to Bevacizumab in GBM patients. We further demonstrated in mouse model that treatment with B20.4.1.1, the mouse analog of Bevacizumab increased macrophage recruitment to the tumor area and correlated with upregulated TNFα expression in GAMs and increased EC activation, which may be a critical molecular mechanism underlying failure of AATx in GBMs. These results suggest TNFα is a novel therapeutic that may reverse resistance to AATx. Future clinical studies should be aimed at inhibiting TNFα as a concurrent therapy in GBMs.

Tumor microenvironment is known to play a critical role in tumorigenesis. Stromal cells can produce a plethora of growth factors, extracellular matrix components, chemokines and angiogenic molecules that can alter the tumor microenvironment and significantly affect the growth and survival of tumor cells [16]. In GBM, the most abundant stromal cell type are macrophages, which can comprise up to 45% of the tumor volume and 80% of GBM associated macrophages are differentiated from recruited bone marrow cells [7, 26]. Here we show that GAMs induce ECs to express an angiogenic gene expression profile with high expression of *VCAM1, ICAM1, CXCL5,* and *CXCL10*. Upregulation of *VCAM1* and *ICAM1* on ECs has been shown to promote further recruitment and attachment of monocytes to the blood vessel wall where they can then migrate into the brain parenchyma, differentiate into macrophages, and exacerbate a GAM-induced inflammatory response [6, 26, 41]. We further showed that expression of VCAM1 is high in high grade gliomas but low or absent in low grade gliomas. Notably, co-staining of VCAM1 and CD31 showed a distinct association of VCAM1 with the pathognomonic ‘glomerular tufts’ in GBM, indicating that neo-vascularized EC are activated. VCAM1 staining is restricted to the outside of vascular EC while CD31 is restricted to the luminal or inner vessel wall. High VCAM1 correlated with poor overall survival, supporting that activated ECs reflect a pro-tumoral and pro-inflammatory state and that VCAM1 serves as a poor prognostic indicator for GBM.

It is very interesting that we demonstrated the upregulation of VCAM1 in the endothelial cells in pathognomonic ‘glomerular tufts’ in GBM. However, the functional significance of VCAM1 expression in the glomeruloid blood vessels is unclear. One possibility is that these events facilitate the entry of additional circulating macrophages that express the VCAM1 counter-receptors alpha4 integrin [2, 8]. Another possibility is that VCAM1 upregulation enables glioma cells to interact with the endothelial cells via alpha4 integrin [36]. To define the functions for VCAM1 in the formation of glomeruloid blood vessels, we performed IHC with alpha4 integrin on tumor sections from the GL261 syngeneic mouse model treated with vehicle control or the TNFα inhibitor MP6-XT22. Alpha4 integrin was strongly expressed in the GL261 mouse tumor microenvironment, including circulating cells (most likely monocytes) in blood vessels which may later exit and differentiate into macrophages (Additional file 1: Figure S5a, arrow). However, when the mice were treated with MP6-XT22, the expression of alpha4 integrin in the tumor microenvironment was dramatically reduced compared to control (Additional file 1: Figure S5a), which is explained by the reduced expression of VCAM1 after treatment with MP6-XT22 as shown in Fig. 5e, f. These data suggests that VCAM1 upregulation enables glioma cells to interact with ECs via alpha4 integrin.

We also performed flow cytometry analysis to determine the expression of alpha4 integrin in RAW264.7 cells and U973 cells, a monocyte cell line derived from a histiocytic lymphoma patient as a positive control. Over 28.5% of RAW264.7 cells express alpha4 integrin (CD49d) while 100% of U973 cells were positive (Additional file 1: Figure S5b).

Although macrophages may provide initial anti-tumor function they can also “re-program” into immune-suppressive, tumor-promoting cells [12]. Given the integral role of macrophages in GBM angiogenesis, novel GAM-targeted therapies may be the key to enhance the efficacy of current therapeutics. Understanding the molecular mechanisms that underlie ‘re-programing’ and polarization switch of macrophages is key for design of effective therapeutics. Here we have shown that glioma cells induce macrophages to secrete TNFα which act on nearby ECs to upregulate genes involved in EC activation. Indeed, TNFα inhibition using the MP6-XT22 antibody significantly increased survival in intracranial tumor-bearing mice and decreased EC activation. Moreover, we provide evidence that TNFα expression can serve as a predictor of response to Bevacizumab in GBM, as patients with high TNFα expression had a shorter survival in response to Bevacizumab compared to those with low TNFα expression. These results demonstrate that TNFα inhibition may be a useful therapeutic strategy in GBM. In addition, GAMs are being group together as likely a heterogeneous population and using mouse-derived cells, individual lineages will be tested in future studies as other myeloid cell populations (including myeloid-derived suppressor cells) have a well-reported function in angiogenesis and can penetrate the tumor microenvironment [37].

With recent development of proteomics techniques, cytokines and other biomarkers can be sensitively detected in liquid biopsy with minimal invasiveness. It has been shown that serum levels of TNFα appeared significantly enhanced in GBM patients [1], suggesting a strong correlation with the disease. The level of TNFα in liquid biopsy may be used to stratify GBM patients before AATx or other adjuvant therapy and follow up them during and after treatments. Further study in correlation of the level of TNFα in liquid biopsy with patient outcome in larger cohort of AATx treated GBM patients will have remarkable implication to these patients.

## Conclusions

In this study, we report a new mechanism of endothelial cell activation in GBM, which is mediated by TNFα secreting GAMs. Inhibition of TNFα blocks GAM-induced EC activation and improve survival in mouse glioma models. Importantly we show that high TNFα expression predicts worse response to Bevacizumab in GBM patients. We further demonstrated in mouse model that treatment with B20.4.1.1, the mouse analog of Bevacizumab increased macrophage recruitment to the tumor area and correlated with upregulated TNFα expression in GAMs and increased EC activation, which may be responsible for the failure of AATx in GBMs. These results suggest TNFα is a novel therapeutic that may reverse resistance to AATx. Future clinical studies should be aimed at inhibiting TNFα as a concurrent therapy in GBMs.

## Supplementary Information

**Additional file 1.** **Figure S1**. **RT-PCR angiogenesis array** Human umbilical vein cells (HUVEC) were incubated with media alone (EBM) or conditioned medium (CM) from RAW264.7 macrophages stimulated with normal human astrocytes (Mφ-NHA) or GBM cell lines U87, U118, U251, A172 (Mφ-U87, Mφ-U118, Mφ-U251, Mφ-A172). mRNA was extracted and analyzed by an RT-PCR angiogenesis array. Results were normalized to RPL30 and ACTB. The relative quantity of significantly altered genes (> 2-fold relative to Mφ-NHA or Mφ-NSC) are shown. n = 3, mean ± max/min, 95% confidence interval. **Figure S2**. **Non-Glioma associated macrophages do not activate ECs** Medium alone (EBM) or CM from normal human astrocytes (Mφ-NHA), GBM cell line (Mφ-U87), renal cell adenocarcinoma cell line (Mφ-786-O), human non-small cell carcinoma cell line (Mφ-H1299), transformed Schwanoma cell line (Mφ-Hei193) or osteosarcoma cell line (Mφ-U2OS) were analyzed by a multi-analyte inflammatory ELISA array. Only the U87 GBM cell line induces upregulation of *VCAM1*, *ICAM1*, *CXCL5* and *CXCL10*. n = 3, mean ± max/min, 95% confidence interval. *p < 0.05. **Figure S3**. **RT-PCR angiogenesis array** Human cerebral ECs (hCMEC/D3) were incubated with media alone (EBM) or conditioned medium (CM) from RAW264.7 macrophages stimulated with normal human astrocytes (Mφ-NHA) or GBM cell lines U87 (Mφ-U87). mRNA was extracted and analyzed by an RT-PCR angiogenesis array. Results were normalized to RPL30 and ACTB. The relative quantity of significantly altered genes are shown. n = 3, mean ± max/min, 95% confidence interval. **Figure S4**. **VEGF RT-PCR assay** Human umbilical vein cells (HUVEC) were incubated with media alone (EBM) or conditioned medium (CM) from RAW264.7 macrophages stimulated with normal mouse astrocytes (Mφ-NMA) or GL-261 cell lines (Mφ-GL261). mRNA was extracted and analyzed by an RT-PCR with VEGF gene. Results were normalized to RPL30 and ACTB. The relative quantity of VEGF is shown (n=3, mean ± max/min, 95% confidence interval). **Figure S5**. **Expression of alpha 4 integrin in tumor microenvironment and on cells** **a**) IHC with alpha 4 integrin has been performed on tumor sections from GL261 syngeneic mouse model treated with vehicle control or TNFα inhibitor, MP6-XT22. **b**) Flow cytometry analysis todetermine the expression of alpha 4 integrin on Raw264.7 cells and U973 cells as a positive control. Anti-Integrin alpha 4/CD49D antibody (ab202969) was diluted in 1:100 for both IHC and Flow cytometry. Alexa 488 donkey anti-rabbit secondary antibody (A32790) was diluted in 1:1000.

## Data Availability

The datasets used and/or analyzed during the current study available from the corresponding author on reasonable request.

## Abbreviations

GBM: Glioblastoma
GAM: Glioma-associated macrophages
EC: Endothelial cells
AATx: Anti-angiogenic therapy
VEGF: Vascular endothelial growth factor
DMEM: Dulbecco’s modified Eagle’s medium
FBS: Fetal bovine serum
NHA: Normal human astrocytes
hNSC-h9: Human neural stem cells-H9 derived
GSC: Glioma stem cells
HUVEC: Human umbilical vein endothelial cells
EGM-2: Endothelial growth media 2
hCMEC/D3: Human cerebral endothelial cell line D3
NCI: National Cancer Institute
RPMI: Roswell Park Memorial Institute medium
NMA: Normal mouse astrocytes
NEAA: Non-Essential Amino Acids
GAM-CM: Glioma-associated macrophage conditioned medium
SDS-PAGE: Sodium dodecyl sulfate–polyacrylamide gel electrophoresis
PVDF: Polyvinylidene difluoride
FFPE: Formalin-fixed paraffin-embedded
GDC: Genomic Data Commons
OS: Overall survival
IDH-wt: IDH-wildtype
IHC: Immunohistochemical
HR: Hazard ratio
CI: Confidence interval
ANOVA: Analysis of variance
LGG: Lower grade
IL8: Secrete interleukin 8
CCL2: Chemokine (C–C motif) ligand 2
F3: Coagulation factor III
FACS: Fluorescence-activated cell sorting
