# Supplementary material for: TNFα secreted by glioma associated macrophages promotes endothelial activation and resistance against anti-angiogenic therapy
Source: Acta Neuropathol Commun. 2021 Apr 14;9:67. doi: 10.1186/s40478-021-01163-0 (PMC8048292; doi:10.1186/s40478-021-01163-0)
Supplement: Supplementary file 1 — Additional file 1. Figure S1. RT-PCR angiogenesis array Human umbilical vein cells (HUVEC) were incubated with media alone (EBM) or conditioned medium (CM) from RAW264.7 macrophages stimulated with normal human astrocytes (Mφ-NHA) or GBM cell lines U87, U118, U251, A172 (Mφ-U87, Mφ-U118, Mφ-U251, Mφ-A172). mRNA was extracted and analyzed by an RT-PCR angiogenesis array. Results were normalized to RPL30 and ACTB. The relative quantity of significantly altered genes (> 2-fold relative to Mφ-NHA or Mφ-NSC) are shown. n = 3, mean ± max/min, 95% confidence interval. Figure S2. Non-Glioma associated macrophages do not activate ECs Medium alone (EBM) or CM from normal human astrocytes (Mφ-NHA), GBM cell line (Mφ-U87), renal cell adenocarcinoma cell line (Mφ-786-O), human non-small cell carcinoma cell line (Mφ-H1299), transformed Schwanoma cell line (Mφ-Hei193) or osteosarcoma cell line (Mφ-U2OS) were analyzed by a multi-analyte inflammatory ELISA array. Only the U87 GBM cell line induces upregulation of VCAM1, ICAM1, CXCL5 and CXCL10. n = 3, mean ± max/min, 95% confidence interval. *p < 0.05. Figure S3. RT-PCR angiogenesis array Human cerebral ECs (hCMEC/D3) were incubated with media alone (EBM) or conditioned medium (CM) from RAW264.7 macrophages stimulated with normal human astrocytes (Mφ-NHA) or GBM cell lines U87 (Mφ-U87). mRNA was extracted and analyzed by an RT-PCR angiogenesis array. Results were normalized to RPL30 and ACTB. The relative quantity of significantly altered genes are shown. n = 3, mean ± max/min, 95% confidence interval. Figure S4. VEGF RT-PCR assay Human umbilical vein cells (HUVEC) were incubated with media alone (EBM) or conditioned medium (CM) from RAW264.7 macrophages stimulated with normal mouse astrocytes (Mφ-NMA) or GL-261 cell lines (Mφ-GL261). mRNA was extracted and analyzed by an RT-PCR with VEGF gene. Results were normalized to RPL30 and ACTB. The relative quantity of VEGF is shown (n=3, mean ± max/min, 95% confidence interval). Figure S5. [file 40478_2021_1163_MOESM1_ESM.docx]

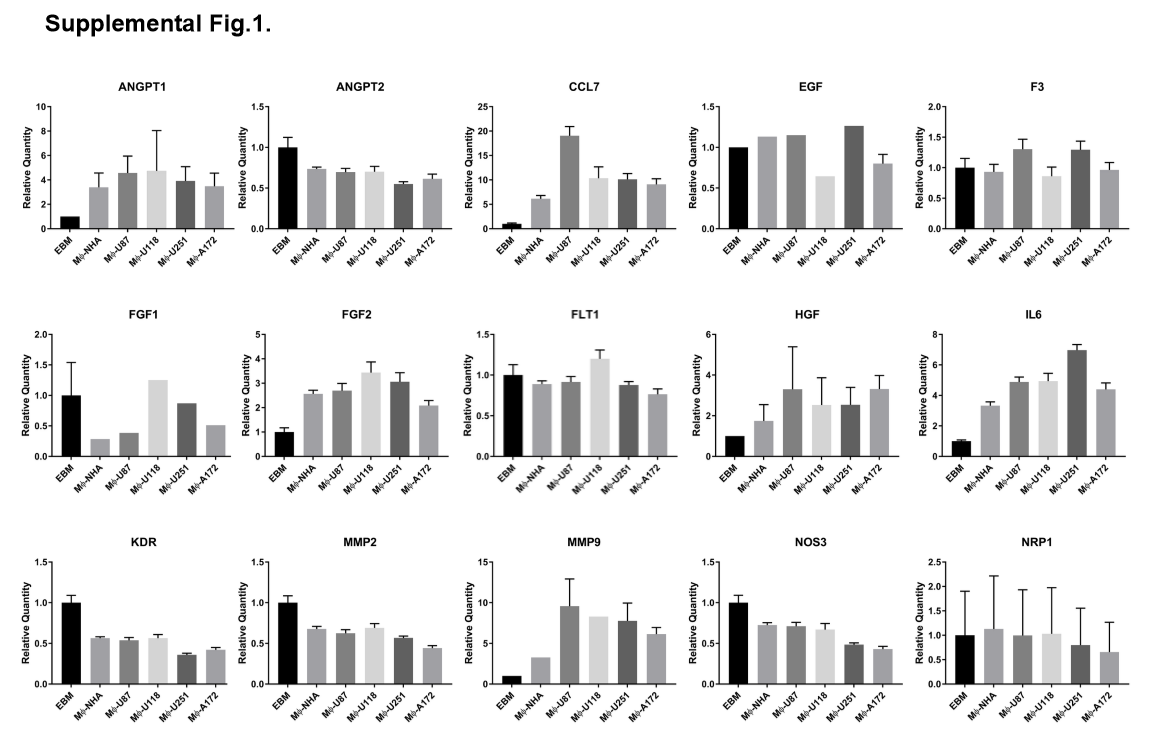

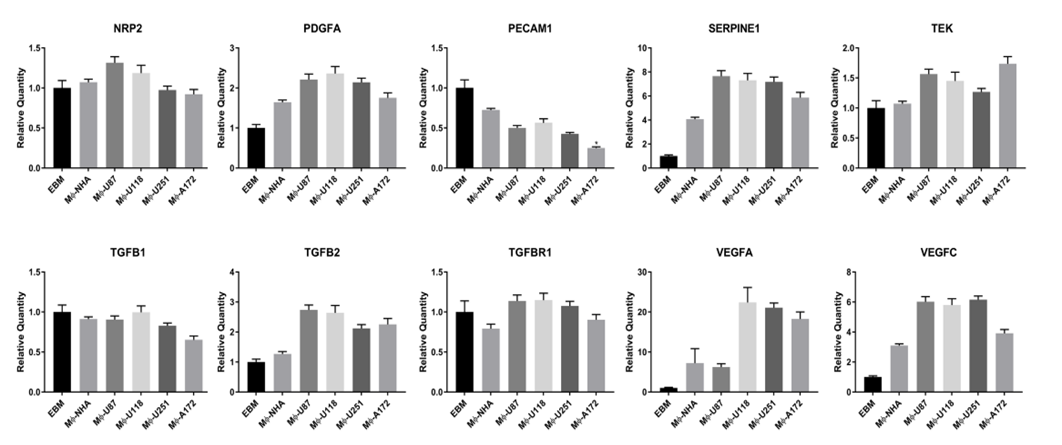


**Figure S1**. **RT-PCR angiogenesis array** Human umbilical vein cells (HUVEC) were incubated with media alone (EBM) or conditioned medium (CM) from RAW264.7 macrophages stimulated with normal human astrocytes (Mφ-NHA) or GBM cell lines U87, U118, U251, A172 (Mφ-U87, Mφ-U118, Mφ-U251, Mφ-A172). mRNA was extracted and analyzed by an RT-PCR angiogenesis array. Results were normalized to *RPL30* and *ACTB*. The relative quantity of significantly altered genes (>2-fold relative to Mφ-NHA or Mφ-NSC) are shown. n=3, mean ± max/min, 95% confidence interval.

**
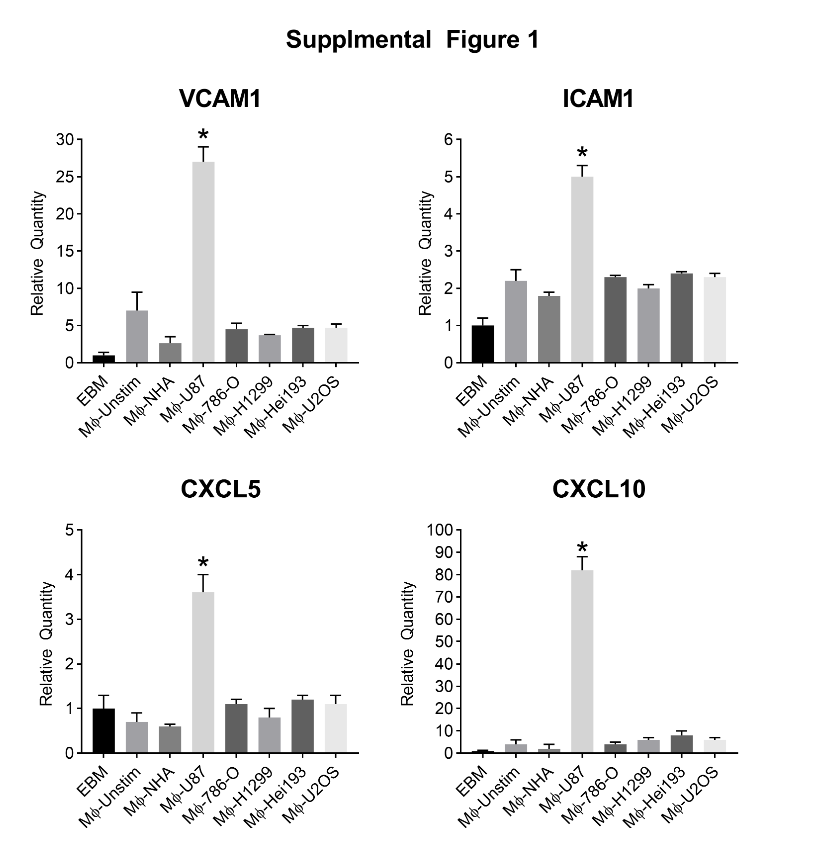
**

**Figure S2**. **Non-Glioma associated macrophages do not activate ECs** Medium alone (EBM) or CM from normal human astrocytes (Mφ-NHA), GBM cell line (Mφ-U87), renal cell adenocarcinoma cell line (Mφ-786-O), human non-small cell carcinoma cell line (Mφ-H1299), transformed Schwanoma cell line (Mφ-Hei193) or osteosarcoma cell line (Mφ-U2OS) were analyzed by a multi-analyte inflammatory ELISA array. Only the U87 GBM cell line induces upregulation of *VCAM1*, *ICAM1*, *CXCL5* and *CXCL10*. n=3, mean ± max/min, 95% confidence interval. *p < 0.05.

**
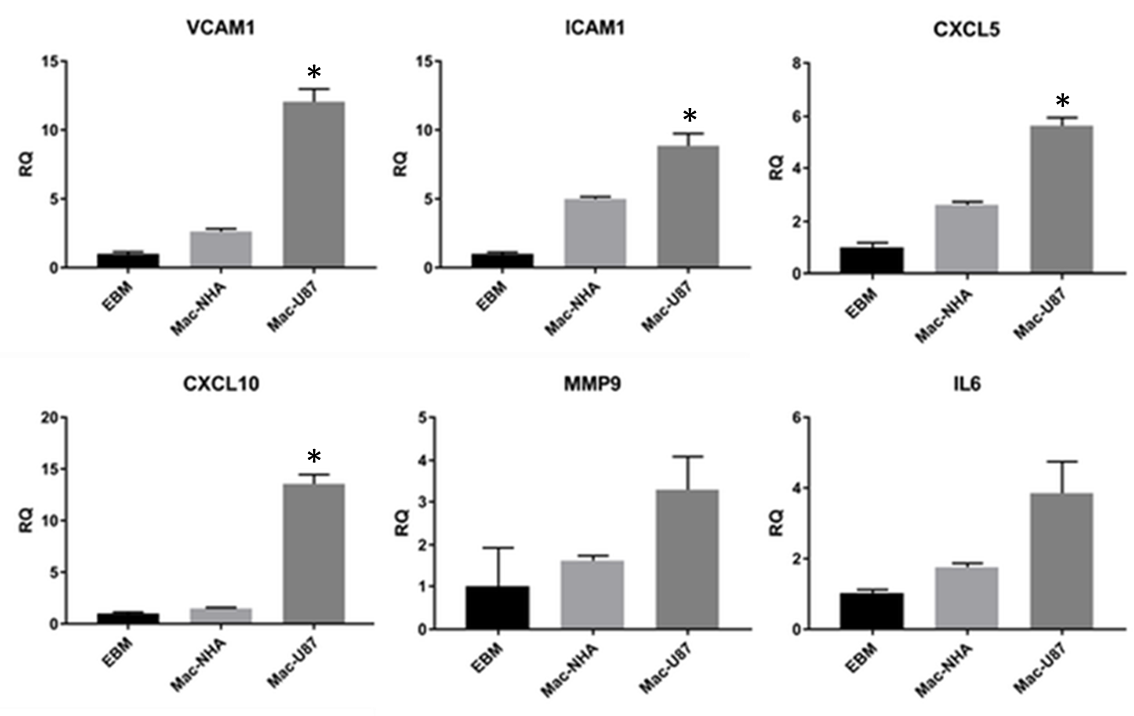
**

**Figure S3. RT-PCR angiogenesis array** Human cerebral ECs (hCMEC/D3) were incubated with media alone (EBM) or conditioned medium (CM) from RAW264.7 macrophages stimulated with normal human astrocytes (Mφ-NHA) or GBM cell lines U87 (Mφ-U87). mRNA was extracted and analyzed by an RT-PCR angiogenesis array. Results were normalized to RPL30 and ACTB. The relative quantity of significantly altered genes are shown. n=3, mean ± max/min, 95% confidence interval.


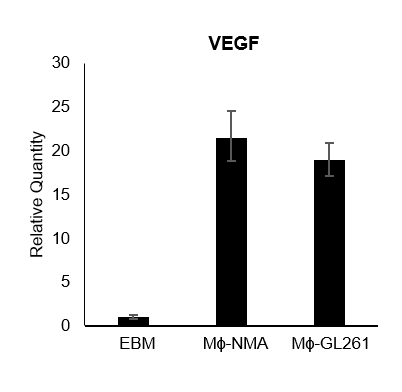


**Figure S4. VEGF RT-PCR** **assay** Human umbilical vein cells (HUVEC) were incubated with media alone (EBM) or conditioned medium (CM) from RAW264.7 macrophages stimulated with normal mouse astrocytes (Mφ-NMA) or GL-261 cell lines (Mφ-GL261). mRNA was extracted and analyzed by an RT-PCR with VEGF gene. Results were normalized to RPL30 and ACTB. The relative quantity of VEGF is shown (n=3, mean ± max/min, 95% confidence interval).


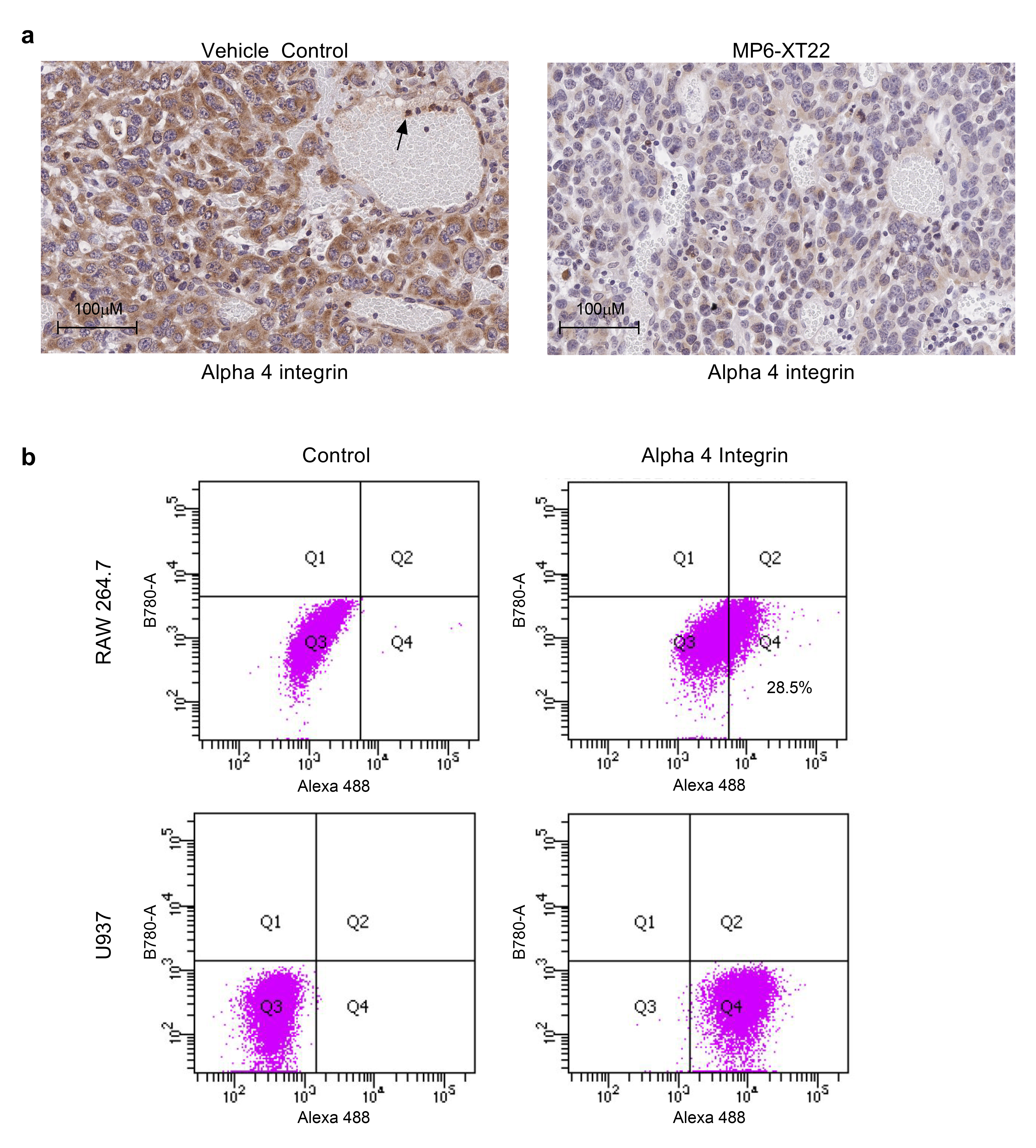


**Figure S5**. Expression of alpha 4 integrin in tumor microenvironment and on cells. **a**) IHC with alpha 4 integrin has been performed on tumor sections from GL261 syngeneic mouse model treated with vehicle control or TNFα inhibitor, MP6-XT22. **b)** Flow cytometry analysis to determine the expression of alpha 4 integrin on Raw264.7 cells and U973 cells as a positive control. Anti-Integrin alpha 4/CD49D antibody (ab202969) was diluted in 1:100 for both IHC and Flow cytometry. Alexa 488 donkey anti-rabbit secondary antibody (A32790) was diluted in 1:1000.

**
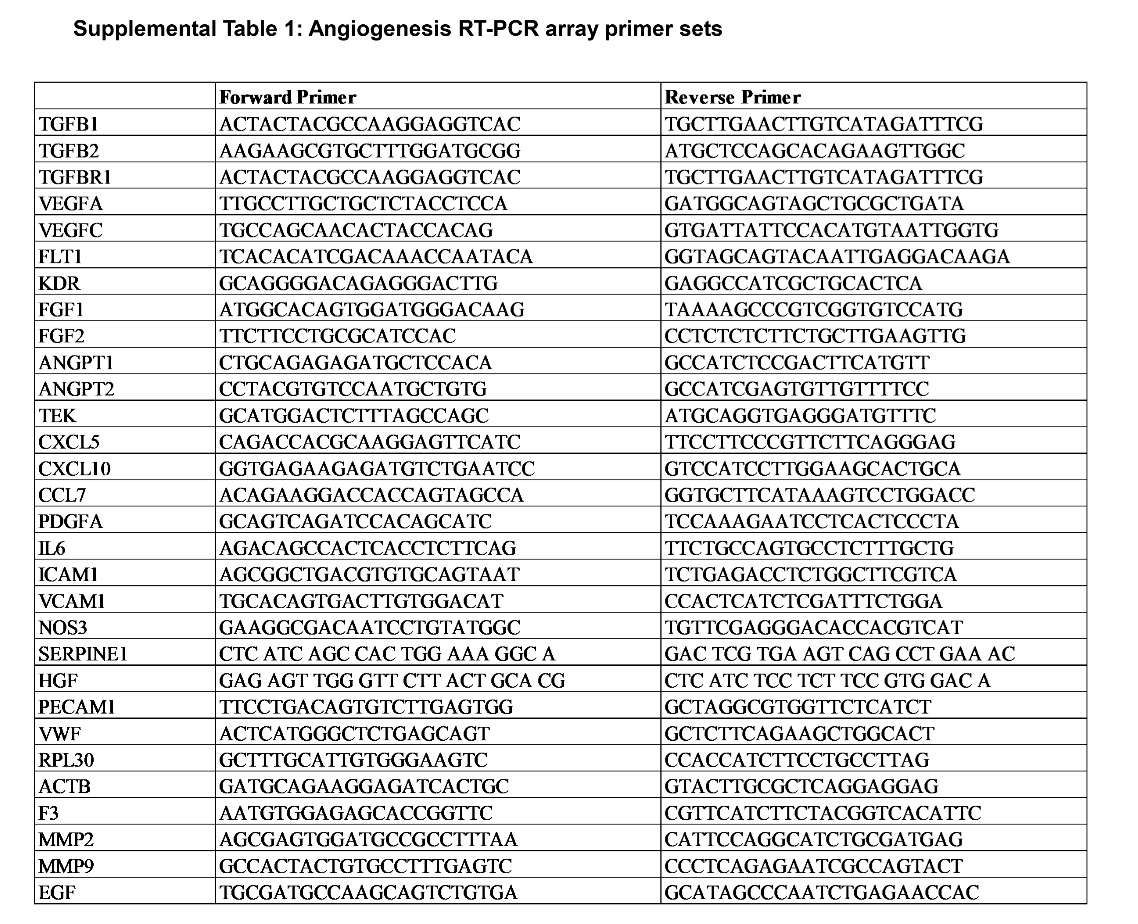
**
